# A Novel Interval-Valued q-Rung Dual Hesitant Linguistic Multi-Attribute Decision-Making Method Based on Linguistic Scale Functions and Power Hamy Mean

**DOI:** 10.3390/e24020166

**Published:** 2022-01-22

**Authors:** Xiaopu Shang, Xue Feng, Jun Wang

**Affiliations:** 1 School of Economics and Management, Beijing Jiaotong University, Beijing 100044, China; sxp@bjtu.edu.cn (X.S.); 19120604@bjtu.edu.cn (X.F.); 2 Beijing Logistics Informatics Research Base, Beijing 100044, China; 3 School of Economics and Management, Beijing University of Chemical Technology, Beijing 100029, China

**Keywords:** interval-valued q-rung dual hesitant linguistic set, linguistic scale function, power Hamy mean operator, multi-attribute decision-making

## Abstract

The interval-valued q-rung dual hesitant linguistic (IVq-RDHL) sets are widely used to express the evaluation information of decision makers (DMs) in the process of multi-attribute decision-making (MADM). However, the existing MADM method based on IVq-RDHL sets has obvious shortcomings, i.e., the operational rules of IVq-RDHL values have some weaknesses and the existing IVq-RDHL aggregation operators are incapable of dealing with some special decision-making situations. In this paper, by analyzing these drawbacks, we then propose the operations for IVq-RDHL values based on a linguistic scale function. After it, we present novel aggregation operators for IVq-RDHL values based on the power Hamy mean and introduce the IVq-RDHL power Hamy mean operator and IVq-RDHL power weighted Hamy mean operator. Properties of these new aggregation operators are also studied. Based on these foundations, we further put forward a MADM method, which is more reasonable and rational than the existing one. Our proposed method not only provides a series of more reasonable operational laws but also offers a more powerful manner to fuse attribute values. Finally, we apply the new MADM method to solve the practical problem of patient admission evaluation. The performance and advantages of our method are illustrated in the comparative analysis with other methods.

## 1. Introduction

Multi-attribute decision-making (MADM) is a frequently used method for determining choices in daily life. This is because most real-life decision-making problems are very complicated, and to make a wise decision, decision makers (DMs) have to evaluate all the feasible forms of multiple aspects before determining the ranking order of alternatives [1,2,3,4,5,6,7,8,9,10]. There are many methods to obtain the rankings of alternatives in the MADM framework, and the aggregation operator (AO) is a powerful technique, which helps DMs acquire the optimal or best alternative. AO refers to a series of special functions that integrate individual attribute values into a collective one. By ranking the comprehensive evaluation values of candidates, the corresponding ranking results of alternatives are determined. More and more complex realistic decision-making problems have prompted many scholars and scientists to realize the importance of studying and exploring the interaction between attributes. This is because there exist complex interrelationships between attributes, which should be considered along with the attribute values when obtaining the comprehensive evaluation ranks of alternatives. Considering it, the Bonferroni mean (BM) [11] and HEronian mean (HEM) [12] were proposed to capture the interrelationship among interacted attributes. Therefore, BM and HEM were widely used by scholars to deal with MADM problems [13,14,15,16] to produce reasonable aggregation results. However, it is insufficient to solely use BM or HEM to aggregate attributes, because DMs’ may provide extreme evaluation values and negatively affect the decision-making results. Due to the lack of enough professional knowledge and time shortage, the decision experts may put forward some extreme values subjectively, which can have a negative influence on aggregation results. He et al. [17] noticed this phenomenon, and they combined the power average (PA) [18] with BM and proposed the power Bonferroni mean (PBM) operator. The authors applied PBM in hesitant fuzzy sets and comparative analysis reveals the advantages of PBM over PA and BM [17]. Afterwards, Liu and Li [19] soon extended PBM to interval-valued intuitionistic fuzzy sets, proposed interval-valued intuitionistic fuzzy AOs and applied them in an air quality evaluation in Guangzhou, China. Wang and Li [20] studied PBM in a Pythagorean fuzzy environment, along with the interactive operational rules. Under the framework of the Dempster–Shafer theory, Liu and Gao [21] continued to study intuitionistic fuzzy PBM operators and applied them in air-condition systems selection. Liu and Liu [22] further generalized PBM into linguistic intuitionistic fuzzy sets and investigated their applications in MADM problems. Liu and Liu [23] used PBM to fuse linguistic q-rung orthopair fuzzy information. He et al. [24] studied the dual form of PBM, proposed the power geometric BM and investigated its performance and advantages in MADM problems. Similar to the PBM operator, Liu [25] introduced the definition of power HEM (PHEM) operator by integrating PA with HEM. PHEM has similar performance and capability as PBM, and it has also been investigated under hesitant fuzzy linguistic terms sets [26], linguistic neutrosophic sets [27], q-rung orthopair hesitant fuzzy sets [28], neutrosophic cubic sets [29], interval-valued dual hesitant fuzzy sets [30], etc., which illustrates the effectiveness and advantages of PHEM.

The aforementioned literature implies the high efficiency of PBM and PHEM in aggregating fuzzy decision-making information under different environments; however, the shortcoming of PBM and PHEM is still prominent, i.e., they just consider the interrelationship that exists between the two attributes rather than among all attributes. Later, Liu et al. [31] proposed the power Hamy mean (PHM) operators, with a combination of PA with Hamy mean (HM) [32] and studied it in interval neutrosophic MADM problems. Compared with PBM and PHEM, PHM takes the interrelationship among multiple input arguments into consideration, and this feature makes PHM suitable and flexible to be employed to fuse attribute values. Based on the pioneering works, Liu and Li [33] and Liu et al. [34] utilized PHM to fuse fuzzy linguistic information.

Nevertheless, as practical MADM problems are very complicated, the interval-valued q-rung dual hesitant linguistic (IVq-RDHL) set proposed by Feng and her colleagues [35] can express complex decision information and is a promising information description tool. Therefore, we analyze the advantages and superiorities of IVq-RDHL sets from three perspectives. First of all, more and more scholars have become aware of the importance of allowing DMs to express their assessments quantificationally and qualitatively [36,37,38,39], while IVq-RDHL sets describe both DMs’ quantitative and qualitative evaluation information, providing DMs with an effective manner to convey their ideas. Secondly, IVq-RDHL sets allow the existence of multiple membership degrees (MDs) and non-membership degrees (NMDs), which can effectively express the hesitation of DMs. Thirdly, IVq-RDHL sets can provide more sufficient space for the description of decision information because they allow the sum of the *q*th power of MD and the *q*th power of NMD to be less than or equal to one. In order to make full use of IVq-RDHL sets in decision-making problems, Feng et al. [35] continued to propose operational rules, a comparison method, and AOs of Ivq-RDHL values, and based on which, a novel MADM method was designed. In addition, the performance and advantages of this method proposed are further proved by illustrative examples and comparative analysis.

However, the method proposed by Feng et al. [35] still has some significant shortcomings, which are mainly divided into two aspects. First, the operational laws of IVq-RDHL values cannot solve the different translation requirements of different DMs for the same semantics. (In Section 3, we analyzed the shortcomings of these operating rules in detail.) Second, the method based on IVq-RDHL Maclaurin symmetric mean operators fails to consider the impact of DMs’ unreasonable evaluation value on the final decision result, although they have the capacity of capturing the interrelationship among attributes. More and more evidence shows that while considering the impact of DMs’ evaluation value, the relationship among attributes can also be considered [40,41,42,43,44,45].

Based on the above analysis, an MADM method under IVq-RDHL sets is proposed in this paper. The main motivations of this study are as follows:

(1) Firstly, we introduce the operational laws of IVq-RDHL values based on linguistic scale functions to overcome the shortcoming of existing operations rules. (We analyzed in detail why novel operational rules are more effective and rational in Section 3).
(2) Secondly, considering the good performance of PHM in fusing fuzzy information, we generalized PHM into IVq-RDHL sets and presented AOs for IVq-RDHL values that can overcome the shortcomings of existing AOs.
(3) Thirdly, we introduced a new MADM method under the IVq-RDHL environment based on the novel AOs. In addition, a practical example about the patient admission evaluation is employed to show the validity and advantages of our new method.

The paper is structured as follows. We review basic notions connected with IVq-RDHL sets in Section 2. Section 3 proposes novel operations for IVq-RDHL values based on a linguistic scale function and studies their properties. Section 4 puts forward new IVq-RDHL AOs based on PHM. Section 5 introduces a new IVq-RDHL MADM method. Section 6 is a series of illustrative examples. Section 7 summarizes the manuscript and outlines future research.

## 2. Preliminaries

In this section, we briefly review the basic concepts, including IVq-RDHLSs, Hamy mean, power average, and power Hamy mean operators.

### 2.1. The IVq-RDHLSs

**Definition** **1** ([35])**.** *Let* X *be a fixed set and* $\tilde{S}$ *be a continuous linguistic term sets (LTS) of* $S=\left\{s_{i}|i=1,2,\ldots ,t\right\}$*, then an interval-valued q-rung dual hesitant linguistic set (IVq-RDHLS) D defined on* X *is expressed as*

(1) $$D=\left\{\langle x,s_{\theta \left(x\right)},h_{D}\left(x\right),g_{D}\left(x\right)\rangle |x\in X\right\},$$

*where* $s_{\theta \left(x\right)}\in \tilde{S}$ *,* $h_{D}\left(x\right)=\cup_{\left[r_{D}^{l},r_{D}^{u}\right]\in h_{D}\left(x\right)}\left\{\left[r_{D}^{l},r_{D}^{u}\right]\right\}$ *and* $g_{D}\left(x\right)=\cup_{\left[\eta_{D}^{l},\eta_{D}^{u}\right]\in g_{D}\left(x\right)}\left\{\left[\eta_{D}^{l},\eta_{D}^{u}\right]\right\}$ *are two sets of interval values in [0, 1], denoting the possible MG and NMG of the element.* x∈X *to the set* D*, respectively, such that* $\left[r_{D}^{l},r_{D}^{u}\right],\left[\eta_{D}^{l},\eta_{D}^{u}\right]\in \left[0,1\right]$*, and* $0\leq {\left({\left(r_{D}^{u}\right)}^{+}\right)}^{q}+{\left({\left(\eta_{D}^{u}\right)}^{+}\right)}^{q}\leq 1$ *,* q≥1, *where,* $\left[\eta_{D}^{l},\eta_{D}^{u}\right]\in g_{D}\left(x\right)$*,* ${\left(r_{D}^{u}\right)}^{+}\in h_{D}^{+}\left(x\right)=\cup_{\left[r_{D}^{l},r_{D}^{u}\right]\in h_{D}\left(x\right)}\max\left\{r_{D}^{u}\right\}$, *and* ${\left(\eta_{D}^{u}\right)}^{+}\in g_{D}^{+}\left(x\right)=\cup_{\left[\eta_{D}^{l},\eta_{D}^{u}\right]\in g_{D}\left(x\right)}\max\left\{\eta_{D}^{u}\right\}$ *for all* x∈X. *For convenience, we call* $d=\langle s_{\theta \left(x\right)},\left\{\left\{\left[r_{D}^{l},r_{D}^{u}\right]\right\},\left\{\left[\eta_{D}^{l},\eta_{D}^{u}\right]\right\}\right\}\rangle$ *an interval-valued q-rung dual hesitant linguistic variable (IVq-RDHLV), which can be denoted as* $d=\langle s_{\theta },\left(h,g\right)\rangle$ *for simplicity. In addition, IVq-RDHLV can transferred into other fuzzy sets. For example, if* q=1, *then D is IVDHFLS; if* q=2*, then D can be simplified to a dual hesitant interval-valued Pythagorean linguistic set; if* $r^{l}=r^{u}$ *and* $\eta^{l}=\eta^{u}$, *then D is simplified to the q-rung dual hesitation linguistic set.*

Then, the operational laws of IVq-RDHLVs are as follows.

**Definition** **2** ([35])**.** *Let* $d=\langle s_{\theta },\left(h,g\right)\rangle$*,* $d_{1}=\langle s_{\theta_{1}},\left(h_{1},g_{1}\right)\rangle$ *and* $d_{2}=\langle s_{\theta_{2}},\left(h_{2},g_{2}\right)\rangle$ *be any three IVq-RDHLVs, then *

*(1)*  $d_{1}⊕d_{2}=\langle s_{\theta_{1}+\theta_{2}},\cup_{r_{1}\in h_{1},r_{2}\in h_{2},\eta_{1}\in g_{1},\eta_{2}\in g_{2}}$
  $$\left\{\left\{\left[{\left({\left(r_{1}^{l}\right)}^{q}+{\left(r_{2}^{l}\right)}^{q}-{\left(r_{1}^{l}r_{2}^{l}\right)}^{q}\right)}^{1/q},{\left({\left(r_{1}^{u}\right)}^{q}+{\left(r_{2}^{u}\right)}^{q}-{\left(r_{1}^{u}r_{2}^{u}\right)}^{q}\right)}^{1/q}\right]\right\},\left\{\left[\eta_{1}^{l}\eta_{2}^{l},\eta_{1}^{u}\eta_{2}^{u}\right]\right\}\right\}\rangle ;$$
*(2)*  $d_{1}⊗d_{2}=\langle s_{\theta_{1}\times \theta_{2}},\cup_{r_{1}\in h_{1},r_{2}\in h_{2},\eta_{1}\in g_{1},\eta_{2}\in g_{2}}$
  $$\left\{\left\{\left[r_{1}^{l}r_{2}^{l},r_{1}^{u}r_{2}^{u}\right]\right\},\left\{\left[{\left({\left(\eta_{1}^{l}\right)}^{q}+{\left(\eta_{2}^{l}\right)}^{q}-{\left(\eta_{1}^{l}\eta_{2}^{l}\right)}^{q}\right)}^{1/q},{\left({\left(\eta_{1}^{u}\right)}^{q}+{\left(\eta_{2}^{u}\right)}^{q}-{\left(\eta_{1}^{u}\eta_{2}^{u}\right)}^{q}\right)}^{1/q}\right]\right\}\right\}\rangle ;$$
*(3)*  $\lambda d=\langle s_{\lambda \theta },\cup_{r\in h,\eta \in g}\left\{\left\{\left[{\left(1-{\left(1-{\left(r^{l}\right)}^{q}\right)}^{\lambda }\right)}^{1/q},{\left(1-{\left(1-{\left(r^{u}\right)}^{q}\right)}^{\lambda }\right)}^{1/q}\right]\right\},\left\{{\left(\eta^{l}\right)}^{\lambda },{\left(\eta^{u}\right)}^{\lambda }\right\}\right\}\rangle$*;*
*(4)*  $d^{\lambda }=\langle s_{\theta^{\lambda }},\cup_{r\in h,\eta \in g}\left\{\left\{{\left(r^{l}\right)}^{\lambda },{\left(r^{u}\right)}^{\lambda }\right\},\left\{\left[{\left(1-{\left(1-{\left(\eta^{l}\right)}^{q}\right)}^{\lambda }\right)}^{1/q},{\left(1-{\left(1-{\left(\eta^{u}\right)}^{q}\right)}^{\lambda }\right)}^{1/q}\right]\right\}\right\}\rangle$.

**Definition** **3** ([35])**.** *Assume that* $d=\langle s_{\theta },\left(h,g\right)\rangle$ *is an IVq-RDHLV, then the score function of d is *

(2) $$S\left(d\right)=\frac{\left(1+{\left(\frac{1}{\#h}\sum_{\left[r^{l},r^{u}\right]\in h}r^{l}\right)}^{q}+{\left(\frac{1}{\#h}\sum_{\left[r^{l},r^{u}\right]\in h}r^{u}\right)}^{q}-{\left(\frac{1}{\#g}\sum_{\left[\eta^{l},\eta^{u}\right]\in g}\eta^{l}\right)}^{q}-{\left(\frac{1}{\#g}\sum_{\left[\eta^{l},\eta^{u}\right]\in g}\eta^{l}\right)}^{q}\right)\times s_{\theta }}{2},$$

*and the accuracy function of d is*

(3) $$p\left(d\right)=\left({\left(\frac{1}{\#h}\sum_{\left[r^{l},r^{u}\right]\in h}r^{l}\right)}^{q}+{\left(\frac{1}{\#h}\sum_{\left[r^{l},r^{u}\right]\in h}r^{u}\right)}^{q}+{\left(\frac{1}{\#g}\sum_{\left[\eta^{l},\eta^{u}\right]\in g}\eta^{l}\right)}^{q}+{\left(\frac{1}{\#g}\sum_{\left[\eta^{l},\eta^{u}\right]\in g}\eta^{l}\right)}^{q}\right)\times s_{\theta },$$

*where* #h *and* #g *represent the number of interval values in* h *and* g, *respectively. For example, assume that* $d=\langle s_{3},\left\{\left\{\left[0.2,0.3\right],\left[0.4,0.6\right]\right\}\left\{\left[0.7,0.8\right]\right\}\right\}\rangle$ *is an IVq-RDHLV. Therefore, we canobtain* #h=2 *and* #g=1.

Suppose that $d_{1}=\langle s_{\theta_{1}},\left(h_{1},g_{1}\right)\rangle$ and $d_{2}=\langle s_{\theta_{2}},\left(h_{2},g_{2}\right)\rangle$ are any two IVq-RDHLVs, then

(1) If $S\left(d_{1}\right)>S\left(d_{2}\right)$, then $d_{1}$ is superior to $d_{2}$, denoted by $d_{1}>d_{2}$;
(2) If $S\left(d_{1}\right)=S\left(d_{2}\right)$, then calculate the accuracy score of the two IVq-RDHLVs
  if $p\left(d_{1}\right)=p\left(d_{2}\right)$, then $d_{1}$ is equivalent to $d_{2}$, denoted by $d_{1}=d_{2}$;
  if $p\left(d_{1}\right)>p\left(d_{2}\right)$, then $d_{1}$ is superior to $d_{2}$, denoted by $d_{1}>d_{2}$.

**Remark** **1.**  *Suppose that there are three IVq-RDHLVs: *$d_{1}=\langle s_{5},\left\{\left\{\left[0.5,0.6\right]\right\},\left\{\left[0.1,0.2\right]\right\}\right\}\rangle$*and *$d_{2}=\langle s_{5},\left\{\left\{\left[0.6,0.7\right]\right\},\left\{\left[0.1,0.2\right],\left[0.3,0.4\right]\right\}\right\}\rangle$, *then the score values can be obtained by the Equation (2), and we have (assume that q = 2)*

$$S\left(d_{1}\right)=\frac{\left(1+{\left(0.5\right)}^{q}+{\left(0.6\right)}^{q}-{\left(0.1\right)}^{q}-{\left(0.2\right)}^{q}\right)\times 5}{2}=\frac{1.56\times 5}{2}=3.9$$

$$S\left(d_{2}\right)=\frac{\left(1+{\left(0.6\right)}^{q}+{\left(0.7\right)}^{q}-{\left(\frac{1}{2}\left(0.1+0.3\right)\right)}^{q}-{\left(\frac{1}{2}\left(0.2+0.4\right)\right)}^{q}\right)\times 5}{2}=\frac{1.72\times 5}{2}=4.3$$

From the results, we can find that $S\left(d_{1}\right)<S\left(d_{2}\right)$, then $d_{1}<d_{2}$. If the score values are the same, then the accuracy values can be considered for comparison, which can be calculated by Equation (3).

### 2.2. HM, PA and PHM Operator

**Definition** **4** ([18])**.** *Suppose that* $a_{i}\left(i=1,2,\ldots ,n\right)$ *is a collection of non-negative crisp numbers, then the PA operator is defined as *

(4) $$PA\left(a_{1},a_{2},\ldots ,a_{n}\right)=\frac{\sum_{i=1}^{n}\left(1+T\left(a_{i}\right)\right)a_{i}}{\sum_{i=1}^{n}\left(1+T\left(a_{i}\right)\right)},$$

*where* $T\left(a_{i}\right)=\sum_{j=1,i\neq j}^{n}Sup\left(a_{i},a_{j}\right)$. *In addition,* $Sup\left(a_{i},a_{j}\right)$ *represents the support for* $a_{i}$ *from* $a_{j}$, *satisfying the conditions:*

*(1)*  $Sup\left(a_{i},a_{j}\right)\in \left[0,1\right]$;

*(2)* 
$Sup\left(a_{i},a_{j}\right)=Sup\left(a_{j},a_{i}\right)$

*(3)*  Sup(a,b)≤Sup(c,d), *if*|a,b|≥|c,d|.

**Definition** **5** ([32])**.** *Assume that* $a_{i}\left(i=1,2,\ldots ,n\right)$ *is a collection of non-negative real numbers. If*

(5) $$HM^{\left(k\right)}\left(a_{1},a_{2},\ldots ,a_{n}\right)=\frac{\sum_{1\leq i_{1}<\cdots <i_{k}\leq n}{\left(\prod_{j=1}^{k}a_{i_{j}}\right)}^{1/k}}{C_{n}^{k}},$$

*then* $HM^{\left(k\right)}$ *is the HM operator. In addition,* k=1,2,…,n*,* $C_{n}^{k}$ *is the binomial coefficient, and* $\left(i_{1},i_{2},\ldots ,i_{k}\right)$ *t* *raverses all the k-tuple combination of* (1,2,…,n).

**Definition** **6** ([31])**.** *Suppose that* $a_{i}\left(i=1,2,\ldots ,n\right)$ *is a collection of non-negative real numbers. Then, the PHM operator is defined as *

(6) $$PHM^{\left(k\right)}\left(a_{1},a_{2},\ldots ,a_{n}\right)=\frac{1}{C_{n}^{k}}\left(\sum_{1\leq i_{1}<\cdots <i_{k}\leq n}{\left(\prod_{j=1}^{k}\left(n\frac{\left(1+T\left(a_{i_{j}}\right)\right)a_{i_{j}}}{\sum_{j=1}^{n}\left(1+T\left(a_{j}\right)\right)}\right)\right)}^{1/k}\right),$$

*where* k=1,2,…,n*,* $C_{n}^{k}$ *is the binomial coefficient, and* $\left(i_{1},i_{2},\ldots ,i_{k}\right)$ *traverses all the k-tuple combination of* (1,2,…,n). *In addition,* $T\left(a_{i}\right)=\sum_{j=1,i\neq j}^{n}Sup\left(a_{i},a_{j}\right)$ *and* $Sup\left(a_{i},a_{j}\right)$ *stand for the support for* $a_{i}$ *from* $a_{j}$ *satisfying the properties presented in Definition 4.*

## 3. Novel Operations of IVq-RDHLVs Based on LSFs

### 3.1. Necessity of Proposing New Operations of IVq-RDHLVs

Feng et al. [35] originated some operations of IVq-RDHLVs, which, however, have some shortcomings.

(1) The existing operations of IVq-RDHLVs are not closed. To illustrate this shortcoming in more detail, we provide the following example.

**Example** **1.** *Assume that there are two IVq-RDHLVs *$a_{1}=\langle s_{5},\left\{\left\{\left[0.3,0.4\right],\left[0.6,0.7\right]\right\},\left\{\left[0.1,0.2\right]\right\}\right\}\rangle$*and *$a_{2}=\langle s_{3},\{\left\{\left[0.4,0.6\right],\left[0.6,0.8\right]\right\}${[0.2,0.5]}}〉*defined on a given LTS *$S=\left\{s_{0},s_{1},\ldots ,s_{5}\right\}$. *According to the operational rules proposed by Feng et al. [35], we have*

*(1)*  $\alpha_{1}⊕\alpha_{2}=\langle s_{8},\left\{\left\{\left[0.4172,0.6139\right],\left[0.6838,0.8488\right]\right\},\left\{\left[0.02,0.1\right]\right\}\right\}\rangle$
*(2)*  $\alpha_{1}⊗\alpha_{2}=\langle s_{15},\left\{\left\{\left[0.12,0.24\right],\left[0.36,0.56\right]\right\},\left\{\left[0.2012,0.5010\right]\right\}\right\}\rangle$
*(3)*  $\alpha_{1}{}^{3}=\langle s_{75},\left\{\left\{\left[0.027,0.064\right],\left[0.216,0.343\right]\right\},\left\{\left[0.1246,0.2491\right]\right\}\right\}\rangle$
*(4)*  $3\alpha_{1}=\langle s_{15},\left\{\left\{\left[0.3735,0.4973\right],\left[0.7358,0.8424\right]\right\},\left\{\left[0.001,0.008\right]\right\}\right\}\rangle$

The calculation result of Example 1 shows that the operation proposed by Feng et al. [35] has a drawback: the calculation results of the linguistic term set (LTS) easily breaks the predefined upper limit; thus, these operations are not closed.

(2) These rules proposed by Feng et al. [35] assumed that the semantic gap between any two adjacent LTs is always equal. However, in practical MADM problems, DMs may feel that the semantic gap will change when the subscript of the LT increase or decrease. For example, DMs may believe that the semantic gap between “extremely poor” and “very poor” is greater or smaller than “good” and “very good”.

### 3.2. The Notion of LFSs

**Definition** **7** ([46])**.** *Assume that* $S=\left\{s_{i}|i=0,1,\ldots ,2t\right\}$ *is an LTS, and* $s_{i}\in S$ *is a linguistic variable. For any real number* $\gamma_{i}\left(i=0,1,2,\ldots ,2t\right)$, *an LSF f is a mapping from* $s_{i}$ *to* $\gamma_{i}\left(i=0,1,2,\ldots ,2t\right)$ *such that:*

(7) $$f:s_{i}\to \gamma_{i}\left(i=0,1,2,\ldots ,2t\right),$$

*where* $0\leq \gamma_{0}<\gamma_{1}<\ldots <\gamma_{2t}$*.*

The three most widely used LSFs are presented as follows.

(8) $$\mathrm{LSF}1:\;f_{1}\left(s_{i}\right)=\gamma_{i}=\frac{i}{2t}\left(i=1,2,\ldots ,2t\right)$$

(9) $$\mathrm{LSF}2:\;f_{2}\left(s_{i}\right)=\gamma_{i}=\left\{ \begin{array}{l} \frac{\rho^{t}-\rho^{t-i}}{2\rho^{t}-2}\;\left(i=0,1,2,\ldots ,t\right)\\ \frac{\rho^{t}+\rho^{t-i}-2}{2\rho^{t-i}-2}\;\left(i=t+1,t+2,\ldots ,2t\right) \end{array}\right.$$

(10) $$\mathrm{LSF}3:\;f_{3}\left(s_{i}\right)=\gamma_{i}=\left\{ \begin{array}{l} \frac{t^{\varepsilon }-{\left(t-i\right)}^{\varepsilon }}{2t^{\varepsilon }}\;\left(i=0,1,2,\ldots ,t\right)\\ \frac{t^{\beta }+{\left(i-t\right)}^{\beta }}{2t^{\beta }}\;\left(i=t+1,t+2,\ldots ,2t\right) \end{array}\right.,\left(\varepsilon ,\beta \in \left[0,1\right]\right),$$

For Equations (9) and (10), it can be seen that the absolute deviations between adjacent linguistic subscripts are different. For example, in Equation (9), the semantic gap between “extremely poor” and “very poor” is smaller than “good” and “very good”. However, in Equation (10), the semantic gap between “extremely poor” and “very poor” is greater than “good” and “very good”. For more information, please refer to the literature by Liu et al. (2019). In particular, if ε=β=1, then Equation (11) is reduced to Equation (8). Meanwhile, the function *f* can be further expanded to a continuous function such that $f*:\tilde{f}\to \Omega^{+}\left(\Omega^{+}=\left\{d|d\geq 0\right\},d\in R\right)$, which satisfies $f*\left(s_{i}\right)=\gamma_{i}$. The inverse function of f* is depicted as $f{*}^{-1}$. Then, we can obtain

(11) $$\mathrm{LSF}\;1:\;f_{1}{*}^{-1}\left(\gamma_{i}\right)=s_{2t*i}\left(i=0,1,2,\ldots ,2t\right),$$

(12) $$\mathrm{LSF}\;2:\;f_{2}{*}^{-1}\left(\gamma_{i}\right)=\left\{ \begin{array}{l} s_{t-\log_{\rho }\left(\rho^{t}-\left(2\rho^{t}-2\right)\gamma_{i}\right)},\;\left(\gamma_{i}\in \left[0,0.5\right]\right)\\ s_{t+\log_{\rho }\left(\left(2\rho^{t}-2\right)\gamma_{i}-\rho^{t}+2\right)},\;\left(\gamma_{i}\in \left[0.5,1.0\right]\right) \end{array}\right.,$$

(13) $$\mathrm{LSF}\;3:\;f_{3}{*}^{-1}\left(\gamma_{i}\right)=\left\{ \begin{array}{l} s_{t-{\left(t^{\varepsilon }-2\times t^{\varepsilon }\times \gamma_{i}\right)}^{1/\varepsilon }},\;\left(\gamma_{\varepsilon }\in \left[0,0.5\right]\right)\\ s_{t+{\left(2\times t^{\beta }\times \gamma_{i}-t^{\beta }\right)}^{1/\beta }},\;\left(\gamma_{\beta }\in \left[0.5,1\right]\right) \end{array}\right.,$$

### 3.3. Operational Rules of IVq-RDHLVs Based on the LSF

**Definition** **8.** *Let* $d=\langle s_{\theta },\left(h,g\right)\rangle$ *,* $d_{1}=\langle s_{\theta_{1}},\left(h_{1},g_{1}\right)\rangle$ *and* $d_{2}=\langle s_{\theta_{2}},\left(h_{2},g_{2}\right)\rangle$ *be any three IVq-RDHLVs, then*

*(1)* $d_{1}⊕d_{2}=\langle f^{*-1}\left(f^{*}\left(\theta_{1}\right)+f^{*}\left(\theta_{2}\right)-f^{*}\left(\theta_{1}\right)f^{*}\left(\theta_{2}\right)\right),$
  $$\cup_{r_{1}\in h_{1},r_{2}\in h_{2},\eta_{1}\in g_{1},\eta_{2}\in g_{2}}\left\{\left\{\left[{\left({\left(r_{1}^{l}\right)}^{q}+{\left(r_{2}^{l}\right)}^{q}-{\left(r_{1}^{l}r_{2}^{l}\right)}^{q}\right)}^{1/q},{\left({\left(r_{1}^{u}\right)}^{q}+{\left(r_{2}^{u}\right)}^{q}-{\left(r_{1}^{u}r_{2}^{u}\right)}^{q}\right)}^{1/q}\right]\right\},\left\{\left[\eta_{1}^{l}\eta_{2}^{l},\eta_{1}^{u}\eta_{2}^{u}\right]\right\}\right\}\rangle ;$$
*(2)* $d_{1}⊗d_{2}=\langle f^{*-1}\left(f^{*}\left(\theta_{1}\right)f^{*}\left(\theta_{2}\right)\right),$
  $$\cup_{r_{1}\in h_{1},r_{2}\in h_{2},\eta_{1}\in g_{1},\eta_{2}\in g_{2}}\left\{\left\{\left[r_{1}^{l}r_{2}^{l},r_{1}^{u}r_{2}^{u}\right]\right\},\left\{\left[{\left({\left(\eta_{1}^{l}\right)}^{q}+{\left(\eta_{2}^{l}\right)}^{q}-{\left(\eta_{1}^{l}\eta_{2}^{l}\right)}^{q}\right)}^{1/q},{\left({\left(\eta_{1}^{u}\right)}^{q}+{\left(\eta_{2}^{u}\right)}^{q}-{\left(\eta_{1}^{u}\eta_{2}^{u}\right)}^{q}\right)}^{1/q}\right]\right\}\right\}\rangle ;$$
*(3)* $\lambda d=\langle f^{*-1}\left(1-{\left(1-f^{*}\left(\theta_{1}\right)\right)}^{\lambda }\right),$
  $$\cup_{r\in h,\eta \in g}\left\{\left\{\left[{\left(1-{\left(1-{\left(r^{l}\right)}^{q}\right)}^{\lambda }\right)}^{1/q},{\left(1-{\left(1-{\left(r^{u}\right)}^{q}\right)}^{\lambda }\right)}^{1/q}\right]\right\},\left\{{\left(\eta^{l}\right)}^{\lambda },{\left(\eta^{u}\right)}^{\lambda }\right\}\right\};$$
*(4)* $d^{\lambda }=\langle f^{*-1}\left({\left(f^{*}\left(\theta \right)\right)}^{\lambda }\right),$
  $$\cup_{r\in h,\eta \in g}\left\{\left\{{\left(r^{l}\right)}^{\lambda },{\left(r^{u}\right)}^{\lambda }\right\},\left\{\left[{\left(1-{\left(1-{\left(\eta^{l}\right)}^{q}\right)}^{\lambda }\right)}^{1/q},{\left(1-{\left(1-{\left(\eta^{u}\right)}^{q}\right)}^{\lambda }\right)}^{1/q}\right]\right\}\right\}\rangle .$$

**Theorem** **1.** *Let* $d=\langle s_{\theta },\left(h,g\right)\rangle$ *,* $d_{1}=\langle s_{\theta_{1}},\left(h_{1},g_{1}\right)\rangle$ *and* $d_{2}=\langle s_{\theta_{2}},\left(h_{2},g_{2}\right)\rangle$ *be any three IVq-RDHLVs, then*

(14) $$(1)\;d_{1}⊕d_{2}=d_{2}⊕d_{1};$$

(15) $$(2)\;d_{1}⊗d_{2}=d_{2}⊗d_{1};$$

(16) $$(3)\;\lambda \left(d_{1}⊕d_{2}\right)=\lambda d_{1}⊕\lambda d_{2}\left(\lambda \geq 0\right);$$

(17) $$(4)\;\lambda_{1}d⊕\lambda_{2}d=\left(\lambda_{1}⊕\lambda_{2}\right)d\left(\lambda_{1},\lambda_{2}\right)\geq 0;$$

(18) $$(5)\;d_{1}^{\lambda }⊗d_{2}^{\lambda }={\left(d_{1}⊗d_{2}\right)}^{\lambda }\left(\lambda \geq 0\right);$$

(19) $$(6)\;d^{\lambda_{1}}⊗d^{\lambda_{2}}=d^{\lambda_{1}+\lambda_{2}}\left(\lambda_{1},\lambda_{2}\geq 0\right).$$

It is easy to prove Equations (14) and (15), so we omit them here.

**Proof.**  According to the operational rules of IVq-RDHLVs in Definition 8, we can obtain

$$\lambda \left(d_{1}⊕d_{2}\right)=\lambda \langle f^{*-1}\left(1-\left(1-f^{*}\left(\theta_{1}\right)\right)\left(1-f^{*}\left(\theta_{2}\right)\right)\right),\;\cup_{r_{1}\in h_{1},r_{2}\in h_{2},\eta_{1}\in g_{1},\eta_{2}\in g_{2}}\left\{\left\{\left[{\left({\left(r_{1}^{l}\right)}^{q}+{\left(r_{2}^{l}\right)}^{q}-{\left(r_{1}^{l}r_{2}^{l}\right)}^{q}\right)}^{1/q},{\left({\left(r_{1}^{u}\right)}^{q}+{\left(r_{2}^{u}\right)}^{q}-{\left(r_{1}^{u}r_{2}^{u}\right)}^{q}\right)}^{1/q}\right]\right\},\left\{\left[\eta_{1}^{l}\eta_{2}^{l},\eta_{1}^{u}\eta_{2}^{u}\right]\right\}\right\}\rangle \;=\langle f^{*-1}\left({\left(\left(1-f^{*}\left(\theta_{1}\right)\right)\left(1-f^{*}\left(\theta_{2}\right)\right)\right)}^{\lambda }\right),\cup_{r_{1}\in h_{1},r_{2}\in h_{2},\eta_{1}\in g_{1},\eta_{2}\in g_{2}}\;\left\{\left\{\left[{\left(1-{\left(1-{\left(r_{1}^{l}\right)}^{q}\right)}^{\lambda }{\left(1-{\left(r_{2}^{l}\right)}^{q}\right)}^{\lambda }\right)}^{1/q},{\left(1-{\left(1-{\left(r_{1}^{U}\right)}^{q}\right)}^{\lambda }{\left(1-{\left(r_{2}^{U}\right)}^{q}\right)}^{\lambda }\right)}^{1/q}\right]\right\},\left\{\left[{\left(\eta_{1}^{l}\eta_{2}^{l}\right)}^{\lambda },{\left(\eta_{1}^{u}\eta_{2}^{u}\right)}^{\lambda }\right]\right\}\right\}\rangle \;\lambda d_{1}⊕\lambda d_{2}=\langle f^{*-1}\left(1-{\left(1-f^{*}\left(\theta_{1}\right)\right)}^{\lambda }\right),\;\cup_{r_{1}\in h_{1},\eta_{1}\in g_{1}}\left\{\left\{\left[{\left(1-{\left(1-{\left(r_{1}^{l}\right)}^{q}\right)}^{\lambda }\right)}^{1/q},{\left(1-{\left(1-{\left(r_{1}^{u}\right)}^{q}\right)}^{\lambda }\right)}^{1/q}\right]\right\},\left\{{\left(\eta_{1}^{l}\right)}^{\lambda },{\left(\eta_{1}^{u}\right)}^{\lambda }\right\}\right\}\;⊕\langle f^{*-1}\left(1-{\left(1-f^{*}\left(\theta_{2}\right)\right)}^{\lambda }\right),\;\cup_{r_{2}\in h_{2},\eta_{2}\in g_{2}}\left\{\left\{\left[{\left(1-{\left(1-{\left(r_{2}^{l}\right)}^{q}\right)}^{\lambda }\right)}^{1/q},{\left(1-{\left(1-{\left(r_{2}^{u}\right)}^{q}\right)}^{\lambda }\right)}^{1/q}\right]\right\},\left\{{\left(\eta_{2}^{l}\right)}^{\lambda },{\left(\eta_{2}^{u}\right)}^{\lambda }\right\}\right\}\;=\langle f^{*-1}\left(\left(1-{\left(1-f^{*}\left(\theta_{1}\right)\right)}^{\lambda }{\left(1-f^{*}\left(\theta_{2}\right)\right)}^{\lambda }\right)\right),\cup_{r_{1}\in h_{1},r_{2}\in h_{2},\eta_{1}\in g_{1},\eta_{2}\in g_{2}}\;\left\{\left\{\left[{\left(1-{\left(1-{\left(r_{1}^{l}\right)}^{q}\right)}^{\lambda }{\left(1-{\left(r_{2}^{l}\right)}^{q}\right)}^{\lambda }\right)}^{1/q},{\left(1-{\left(1-{\left(r_{1}^{U}\right)}^{q}\right)}^{\lambda }{\left(1-{\left(r_{2}^{U}\right)}^{q}\right)}^{\lambda }\right)}^{1/q}\right]\right\},\left\{\left[{\left(\eta_{1}^{l}\eta_{2}^{l}\right)}^{\lambda },{\left(\eta_{1}^{u}\eta_{2}^{u}\right)}^{\lambda }\right]\right\}\right\}\rangle \;=\lambda \left(d_{1}⊕d_{2}\right)$$

According to the above process, it is obvious that Equation (16) holds for λ≥0. In addition, we have

$$\lambda_{1}d⊕\lambda_{2}d=\langle f^{*-1}\left(1-{\left(1-f^{*}\left(\theta \right)\right)}^{\lambda_{1}}{\left(1-f^{*}\left(\theta \right)\right)}^{\lambda_{2}}\right),\cup_{r\in h,\eta \in g}\;\{\left\{\left[{\left(1-{\left(1-{\left(r^{l}\right)}^{q}\right)}^{\lambda_{1}}{\left(1-{\left(r^{l}\right)}^{q}\right)}^{\lambda_{2}}\right)}^{1/q},{\left(1-{\left(1-{\left(r^{U}\right)}^{q}\right)}^{\lambda_{1}}{\left(1-{\left(r^{U}\right)}^{q}\right)}^{\lambda_{1}}\right)}^{1/q}\right]\right\},\;\left\{\left[{\left(\eta^{l}\right)}^{\lambda_{1}}{\left(\eta^{l}\right)}^{\lambda_{2}},{\left(\eta^{U}\right)}^{\lambda_{1}}{\left(\eta^{U}\right)}^{\lambda_{2}}\right]\right\}\}\rangle \;=\left(\lambda_{1}+\lambda_{2}\right)d$$

According to the above process, Equation (17) is kept. Based on Definition 7 and Definition 8, we have

$$d_{1}^{\lambda }⊗d_{2}^{\lambda }=\langle f^{*-1}\left({\left(f^{*}\left(\theta_{1}\right)f^{*}\left(\theta_{2}\right)\right)}^{\lambda }\right),\cup_{r_{1}\in h_{1},r_{2}\in h_{2},\eta_{1}\in g_{1},\eta_{2}\in g_{2}}\;\left\{\left\{\left[{\left(r_{1}^{l}r_{2}^{l}\right)}^{\lambda },{\left(r_{1}^{u}r_{2}^{u}\right)}^{\lambda }\right]\right\},\left\{\left[{\left(1-{\left(1-{\left(\eta_{1}^{l}\right)}^{q}\right)}^{\lambda }{\left(1-{\left(\eta_{2}^{l}\right)}^{q}\right)}^{\lambda }\right)}^{1/q},{\left(1-{\left(1-{\left(\eta_{1}^{U}\right)}^{q}\right)}^{\lambda }{\left(1-{\left(\eta_{2}^{U}\right)}^{q}\right)}^{\lambda }\right)}^{1/q}\right]\right\}\right\}\rangle \;=d_{1}^{\lambda }⊗d_{2}^{\lambda }\;d^{\lambda_{1}}⊗d^{\lambda_{2}}=\langle f^{*-1}\left({\left(f^{*}\left(\theta \right)\right)}^{\lambda_{1}}{\left(f^{*}\left(\theta_{2}\right)\right)}^{\lambda_{2}}\right),\cup_{r\in h,\eta \in g}\{\left\{\left[{\left(r^{l}\right)}^{\lambda_{1}}{\left(r^{l}\right)}^{\lambda_{2}},{\left(r^{U}\right)}^{\lambda_{1}}{\left(r^{U}\right)}^{\lambda_{2}}\right]\right\},\;\left\{\left[{\left(1-{\left(1-{\left(\eta^{l}\right)}^{q}\right)}^{\lambda_{1}}{\left(1-{\left(\eta^{l}\right)}^{q}\right)}^{\lambda_{2}}\right)}^{1/q},{\left(1-{\left(1-{\left(\eta^{U}\right)}^{q}\right)}^{\lambda_{1}}{\left(1-{\left(\eta^{U}\right)}^{q}\right)}^{\lambda_{1}}\right)}^{1/q}\right]\right\}\}\rangle =d^{\lambda_{1}+\lambda_{2}}.$$

So far, Theorem 1 have been proved.□

### 3.4. Comparison Method of IVq-RDHLVs Based on LSF

Based on LSF *f*, the comparison method of IVq-RDHLVs is put forward as follows.

**Definition** **9.** *Assume that* $d=\langle s_{\theta },\left(h,g\right)\rangle$ *is an IVq-RDHLV, then the score function of* d *can be calculated by*

(20) $$S\left(d\right)=\frac{\left(1+{\left(\frac{1}{\#h}\sum_{\left[r^{l},r^{u}\right]\in h}r^{l}\right)}^{q}+{\left(\frac{1}{\#h}\sum_{\left[r^{l},r^{u}\right]\in h}r^{u}\right)}^{q}-{\left(\frac{1}{\#g}\sum_{\left[\eta^{l},\eta^{u}\right]\in g}\eta^{l}\right)}^{q}-{\left(\frac{1}{\#g}\sum_{\left[\eta^{l},\eta^{u}\right]\in g}\eta^{l}\right)}^{q}\right)\times f^{*}\left(\theta \right)}{2}.$$

*and the accuracy function of* d *can be computed by*

(21) $$p\left(d\right)=\left({\left(\frac{1}{\#h}\sum_{\left[r^{l},r^{u}\right]\in h}r^{l}\right)}^{q}+{\left(\frac{1}{\#h}\sum_{\left[r^{l},r^{u}\right]\in h}r^{u}\right)}^{q}+{\left(\frac{1}{\#g}\sum_{\left[\eta^{l},\eta^{u}\right]\in g}\eta^{l}\right)}^{q}+{\left(\frac{1}{\#g}\sum_{\left[\eta^{l},\eta^{u}\right]\in g}\eta^{l}\right)}^{q}\right)\times f^{*}\left(\theta \right).$$

For any two IVq-RDHLVs $d_{1}=\langle s_{\theta_{1}},\left(h_{1},g_{1}\right)\rangle$ and $d_{2}=\langle s_{\theta_{2}},\left(h_{2},g_{2}\right)\rangle$, the specific comparison rules are presented in Definition 3.

### 3.5. Distance Measure of IVq-RDHLVs

In this section, we propose a new concept of the distance between two IVq-RDHLVs based on the LSF.

**Definition** **10.** *Suppose that *$d_{1}=\langle s_{\theta_{1}},\left(h_{1},g_{1}\right)\rangle$*and *$d_{2}=\langle s_{\theta_{2}},\left(h_{2},g_{2}\right)\rangle$*are any two IVq-RDHLVs, then the distance between*$d_{1}$*and *$d_{2}$*is expressed as*

(22) $$d\left(d_{1},d_{2}\right)=(\frac{1}{2\left(\#h+\#g\right)}(\sum_{i=1}^{\#h}\left(\left|{\left({\left(\gamma_{1}^{L}\right)}_{\sigma \left(i\right)}\right)}^{q}-{\left({\left(\gamma_{2}^{L}\right)}_{\sigma \left(i\right)}\right)}^{q}\right|+\left|{\left({\left(\gamma_{1}^{U}\right)}_{\sigma \left(i\right)}\right)}^{q}-{\left({\left(\gamma_{2}^{U}\right)}_{\sigma \left(i\right)}\right)}^{q}\right|\right)+\;\sum_{j=1}^{\#g}\left(\left|{\left({\left(\eta_{1}^{L}\right)}_{\sigma \left(j\right)}\right)}^{q}-{\left({\left(\eta_{2}^{L}\right)}_{\sigma \left(j\right)}\right)}^{q}\right|+\left|{\left({\left(\eta_{1}^{U}\right)}_{\sigma \left(j\right)}\right)}^{q}-{\left({\left(\eta_{2}^{U}\right)}_{\sigma \left(j\right)}\right)}^{q}\right|\right))\times \left|f^{*}\left(\theta_{1}\right)-f^{*}\left(\theta_{2}\right)\right|.$$

*where *$\left[{\left(\gamma_{1}^{L}\right)}_{\sigma \left(i\right)},{\left(\gamma_{1}^{U}\right)}_{\sigma \left(i\right)}\right]\in h_{1}$*, *$\left[{\left(\gamma_{2}^{L}\right)}_{\sigma \left(i\right)},{\left(\gamma_{2}^{U}\right)}_{\sigma \left(i\right)}\right]\in h_{2}$*, *$\left[{\left(\eta_{1}^{L}\right)}_{\sigma \left(i\right)},{\left(\eta_{1}^{U}\right)}_{\sigma \left(i\right)}\right]\in g_{1}$*, *$\left[{\left(\eta_{2}^{L}\right)}_{\sigma \left(i\right)},{\left(\eta_{2}^{U}\right)}_{\sigma \left(i\right)}\right]$$\in g_{2}$*, *$\left[{\left(\gamma_{1}^{L}\right)}_{\sigma \left(i\right)},{\left(\gamma_{1}^{U}\right)}_{\sigma \left(i\right)}\right]<\left[{\left(\gamma_{1}^{L}\right)}_{\sigma \left(i+1\right)},{\left(\gamma_{1}^{U}\right)}_{\sigma \left(i+1\right)}\right]$*, *$\left[{\left(\gamma_{2}^{L}\right)}_{\sigma \left(i\right)},{\left(\gamma_{2}^{U}\right)}_{\sigma \left(i\right)}\right]<$$\left[{\left(\gamma_{2}^{L}\right)}_{\sigma \left(i+1\right)},{\left(\gamma_{2}^{U}\right)}_{\sigma \left(i+1\right)}\right]$*, *$\left[{\left(\eta_{1}^{L}\right)}_{\sigma \left(i\right)},{\left(\eta_{1}^{U}\right)}_{\sigma \left(i\right)}\right]<\left[{\left(\eta_{1}^{L}\right)}_{\sigma \left(i+1\right)},{\left(\eta_{1}^{U}\right)}_{\sigma \left(i+1\right)}\right]$*and *$\left[{\left(\eta_{2}^{L}\right)}_{\sigma \left(i\right)},{\left(\eta_{2}^{U}\right)}_{\sigma \left(i\right)}\right]<$$\left[{\left(\eta_{2}^{L}\right)}_{\sigma \left(i+1\right)},{\left(\eta_{2}^{U}\right)}_{\sigma \left(i+1\right)}\right]$. *In addition, #h represents the number of interval values contained in h_1_ and h_2_, and #g represents the number of elements that make up g_1_ and g_2_.*

**Remark** **2.**  ([35])**.** *Assume that* $d_{1}=\langle s_{\theta_{1}},\left(h_{1},g_{1}\right)\rangle$ *and* $d_{2}=\langle s_{\theta_{2}},\left(h_{2},g_{2}\right)\rangle$ *are any two IVq-RDHLVs. From Definition 10, it is obvious that* *#h_1_ = #h_2_ and #g_1_ = #g_2_, which means that h_1_ and h_2_ should have the same number of values, and g_1_ and g_2_ should have the same number of values when calculating the distance.* *However, this condition cannot be always satisfied. In order to make the MD and NMD numbers of the two IVq-RDHFEs equal, Feng et al.* [35] *proposed two methods to adjust the IVq-RDHFEs, which fail to satisfy the condition. Inspired by this idea, we extend the shorter IVq-RDHLV to satisfy the condition by adding the largest interval values in MD and NMD, respectively.*

**Example** **2.** *Assume that there are two evaluation values denoted by IVq-RDHLVs, which are *$d_{1}=\left\{s_{3},\left\{\left[0.3,0.4\right]\right\},\left\{\left[0.3,0.5\right],\left[0.4,0.6\right],\left[0.7,0.8\right]\right\}\right\}$*and *$d_{2}=\{s_{5},\left\{\left[0.2,0.3\right],\left[0.4,0.7\right]\right\}${[0.3,0.5],[0.6,0.8]}}*defined on a given LTS *$S=\left\{s_{0},s_{1},s_{2},s_{3},s_{4},s_{5}\right\}$. *For calculation,*$d_{1}$*and *$d_{2}$*can be transformed to*${d^{\prime }}_{1}$*and *${d^{\prime }}_{2}$, *respectively (*q=5*).*

$${d^{\prime }}_{1}=\left\{s_{3},\left\{\left[0.3,0.4\right],\left[0.3,0.4\right]\right\},\left\{\left[0.3,0.5\right],\left[0.4,0.6\right],\left[0.7,0.8\right]\right\}\right\},\;d_{2}{}^{\prime }=\left\{s_{5},\left\{\left[0.2,0.3\right],\left[0.4,0.7\right]\right\},\left\{\left[0.3,0.5\right],\left[0.3,0.5\right],\left[0.6,0.8\right]\right\}\right\}.$$

If we use LSF1, then the distance between $d_{1}$ and $d_{2}$ is

$$d\left(d_{1},d_{2}\right)=\left|\frac{3}{6}-\frac{5}{5}\right|\times (\frac{1}{2\left(2+3\right)}(\left(\left|{\left(0.3\right)}^{q}-{\left(0.2\right)}^{q}\right|+\left|{\left(0.4\right)}^{q}-{\left(0.3\right)}^{q}\right|+\left|{\left(0.3\right)}^{q}-{\left(0.4\right)}^{q}\right|+\left|{\left(0.4\right)}^{q}-{\left(0.7\right)}^{q}\right|\right)+\;\left(\left|{\left(0.3\right)}^{q}-{\left(0.3\right)}^{q}\right|+\left|{\left(0.5\right)}^{q}-{\left(0.5\right)}^{q}\right|+\left|{\left(0.4\right)}^{q}-{\left(0.3\right)}^{q}\right|+\left|{\left(0.6\right)}^{q}-{\left(0.5\right)}^{q}\right|+\left|{\left(0.7\right)}^{q}-{\left(0.6\right)}^{q}\right|+\left|{\left(0.8\right)}^{q}-{\left(0.8\right)}^{q}\right|\right))\;=\frac{1}{3}\times \left(\frac{1}{10}\left({\left(0.4\right)}^{q}+{\left(0.7\right)}^{q}-{\left(0.3\right)}^{q}-{\left(0.2\right)}^{q}\right)+\left({\left(0.7\right)}^{q}+{\left(0.4\right)}^{q}-{\left(0.3\right)}^{q}-{\left(0.5\right)}^{q}\right)\right)\;\;=0.0107$$

For two IVq-RDHLVs $d_{1}$ and $d_{2}$, $d\left(d_{1},d_{2}\right)$ represents the distance between $d_{1}$ and $d_{2}$ and satisfy the following properties:

(1) $d\left(d_{1},d_{2}\right)\in \left[0,1\right]$;
(2) $d\left(d_{1},d_{2}\right)=0$ if and only if $d_{1}=d_{2}$;
(3) $d\left(d_{1},d_{2}\right)=d\left(d_{2},d_{1}\right)$.

## 4. Aggregation Operators of IVq-RDHLVs and Their Properties

### 4.1. The Interval-Valued q-Rung Dual Hesitant Linguistic Power Hamy Mean (IVq-RDHLPHM) Operator

**Definition** **11.**  *Let *$d_{i}\left(i=1,2,\ldots ,n\right)$*be a collection of IVq-RDHLVs, and then tIVq-RDHLPHM operator is defined as*

(23) $$IVq-RDHLPHM^{\left(k\right)}\left(d_{1},d_{2},\ldots ,d_{n}\right)=\frac{1}{C_{n}^{k}}\left(\underset{1\leq i_{1}<\cdots <i_{k}\leq n}{⊕}{\left(\overset{k}{\underset{j=1}{⊗}}\left(\frac{n\left(1+T\left(d_{i_{j}}\right)\right)d_{i_{j}}}{\sum_{i=1}^{n}\left(1+T\left(d_{i}\right)\right)}\right)\right)}^{1/k}\right),$$

*where *k=1,2,…,n*, *$C_{n}^{k}$*is the binomial coefficient and *$\left(i_{1},i_{2},\ldots ,i_{k}\right)$*traverses all the k-tuple combination of *(1,2,…,n). *Moreover, *$T\left(d_{i}\right)=\sum_{j=1,i\neq j}^{n}Sup\left(d_{i},d_{j}\right)$*and *$Sup\left(d_{i},d_{j}\right)$*stand for the support for *$d_{i}$*from *$d_{j}$*and satisfy the conditions presented in Definition 4.*

In order to simplify Equation (22), assume that

(24) $$\sigma_{i}=\frac{1+T\left(d_{i}\right)}{\sum_{i=1}^{n}\left(1+T\left(d_{i}\right)\right)},$$

then Equation (22) can be transformed as

(25) $$IVq-RDHLPHM^{\left(k\right)}\left(d_{1},d_{2},\ldots ,d_{n}\right)=\frac{1}{C_{n}^{k}}(\underset{1\leq i_{1}<\cdots <i_{k}\leq n}{⊕}{\left(\overset{k}{\underset{j=1}{⊗}}\left(n\sigma_{i_{j}}d_{i_{j}}\right))\right)}^{1/k}),$$

where $0\leq \sigma_{i}\leq 1$ and $\sum_{i=1}^{n}\sigma_{i}=1$.

**Theorem** **2.**  *Let* $d_{i}=\langle s_{\theta_{i}},\left(h_{i},g_{i}\right)\rangle \left(i=1,2,\ldots ,n\right)$ *be a collection of IVq-RDHLVs, then the results aggregated by the IVq-RDHLPHM operator is also an IVq-RDHLV and *

(26) $$IVq-RDHLPHM^{\left(k\right)}\left(d_{1},d_{2},\ldots ,d_{n}\right)=\langle f^{*-1}\left(1-\prod_{1\leq i_{1}<\cdots <i_{k}\leq n}{\left(1-\prod_{j=1}^{k}{\left(1-{\left(1-f^{*}\left(\theta_{i_{j}}\right)\right)}^{n\sigma_{i_{j}}}\right)}^{1/k}\right)}^{\frac{1}{C_{n}^{k}}}\right),\cup_{r_{i_{j}}\in h_{i_{j}},\eta_{i_{j}}\in g_{i_{j}}}\;\{\left\{\left[{\left(1-\prod_{1\leq i_{1}<\ldots <i_{k}\leq n}{\left(1-\prod_{j=1}^{k}{\left(1-{\left(1-{\left(\gamma_{i_{j}}^{L}\right)}^{q}\right)}^{n\sigma_{i_{j}}}\right)}^{1/k}\right)}^{\frac{1}{C_{n}^{k}}}\right)}^{1/q},{\left(1-\prod_{1\leq i_{1}<\ldots <i_{k}\leq n}{\left(1-\prod_{j=1}^{k}{\left(1-{\left(1-{\left(\gamma_{i_{j}}^{U}\right)}^{q}\right)}^{n\sigma_{i_{j}}}\right)}^{1/k}\right)}^{\frac{1}{C_{n}^{k}}}\right)}^{1/q}\right]\right\}\;\left\{\left[\prod_{1\leq i_{1}<\ldots <i_{k}\leq n}{\left(1-\prod_{j=1}^{k}{\left(1-{\left(\eta_{i_{j}}^{L}\right)}^{nq\sigma_{i_{j}}}\right)}^{1/k}\right)}^{1/qC_{n}^{k}},\prod_{1\leq i_{1}<\ldots <i_{k}\leq n}{\left(1-\prod_{j=1}^{k}{\left(1-{\left(\eta_{i_{j}}^{U}\right)}^{nq\sigma_{i_{j}}}\right)}^{1/k}\right)}^{1/qC_{n}^{k}}\right]\right\}\}\rangle .$$

**Proof.**  From Definitions 8 and 9, we can obtain

$$n\sigma_{i_{j}}d_{i_{j}}=\langle f^{*-1}\left(1-{\left(1-f^{*}\left(\theta_{i_{j}}\right)\right)}^{n\sigma_{i_{j}}}\right),\;\cup_{r_{i_{j}}\in h_{i_{j}},\eta_{i_{j}}\in g_{i_{j}}}\left\{\left\{\left[{\left(1-{\left(1-{\left(\gamma_{i_{j}}^{L}\right)}^{q}\right)}^{n\sigma_{i_{j}}}\right)}^{1/q},{\left(1-{\left(1-{\left(\gamma_{i_{j}}^{U}\right)}^{q}\right)}^{n\sigma_{i_{j}}}\right)}^{1/q}\right]\right\},\left\{\left[{\left(\eta_{i_{j}}^{L}\right)}^{n\sigma_{i_{j}}},{\left(\eta_{i_{j}}^{U}\right)}^{n\sigma_{i_{j}}}\right]\right\}\right\}\rangle$$

and

$$\overset{k}{\underset{j=1}{⊗}}\left(n\sigma_{i_{j}}d_{i_{j}}\right)=\langle f^{*-1}\left(\prod_{j=1}^{k}\left(1-{\left(1-f^{*}\left(\theta_{i_{j}}\right)\right)}^{n\sigma_{i_{j}}}\right)\right),\;\cup_{r_{i_{j}}\in h_{i_{j}},\eta_{i_{j}}\in g_{i_{j}}}\{\left\{\left[\prod_{j=1}^{k}{\left(1-{\left(1-{\left(\gamma_{i_{j}}^{L}\right)}^{q}\right)}^{n\sigma_{i_{j}}}\right)}^{1/q},\prod_{j=1}^{k}{\left(1-{\left(1-{\left(\gamma_{i_{j}}^{U}\right)}^{q}\right)}^{n\sigma_{i_{j}}}\right)}^{1/q}\right]\right\},\;\left\{\left[{\left(1-\prod_{j=1}^{k}\left(1-{\left(\eta_{i_{j}}^{L}\right)}^{nq\sigma_{i_{j}}}\right)\right)}^{1/q},{\left(1-\prod_{j=1}^{k}\left(1-{\left(\eta_{i_{j}}^{U}\right)}^{nq\sigma_{i_{j}}}\right)\right)}^{1/q}\right]\right\}\}\rangle .$$

Further,

$$\overset{k}{\underset{j=1}{⊗}}{\left(n\sigma_{i_{j}}d_{i_{j}}\right)}^{1/k}=\langle f^{*-1}\left(\prod_{j=1}^{k}{\left(1-{\left(1-f^{*}\left(\theta_{i_{j}}\right)\right)}^{n\sigma_{i_{j}}}\right)}^{1/k}\right),\;\cup_{r_{i_{j}}\in h_{i_{j}},\eta_{i_{j}}\in g_{i_{j}}}\{\left\{\left[\prod_{j=1}^{k}{\left(1-{\left(1-{\left(\gamma_{i_{j}}^{L}\right)}^{q}\right)}^{n\sigma_{i_{j}}}\right)}^{1/kq},\prod_{j=1}^{k}{\left(1-{\left(1-{\left(\gamma_{i_{j}}^{U}\right)}^{q}\right)}^{n\sigma_{i_{j}}}\right)}^{1/kq}\right]\right\},\;\left\{\left[{\left(1-\prod_{j=1}^{k}{\left(1-{\left(\eta_{i_{j}}^{L}\right)}^{nq\sigma_{i_{j}}}\right)}^{1/k}\right)}^{1/q},{\left(1-\prod_{j=1}^{k}{\left(1-{\left(\eta_{i_{j}}^{U}\right)}^{nq\sigma_{i_{j}}}\right)}^{1/k}\right)}^{1/q}\right]\right\}\}\rangle$$

and

$$\underset{1\leq i_{1}<\ldots <i_{k}\leq n}{⊕}{\left(\overset{k}{\underset{j=1}{⊗}}\left(n\sigma_{i_{j}}d_{i_{j}}\right)\right)}^{1/k}=\langle f^{*-1}\left(1-\prod_{1\leq i_{1}<\cdots <i_{k}\leq n}\left(1-\prod_{j=1}^{k}{\left(1-{\left(1-f^{*}\left(\theta_{i_{j}}\right)\right)}^{n\sigma_{i_{j}}}\right)}^{1/k}\right)\right),\cup_{r_{i_{j}}\in h_{i_{j}},\eta_{i_{j}}\in g_{i_{j}}}\;\{\left\{\left[{\left(1-\prod_{1\leq i_{1}<\ldots <i_{k}\leq n}\left(1-\prod_{j=1}^{k}{\left(1-{\left(1-{\left(\gamma_{i_{j}}^{L}\right)}^{q}\right)}^{n\sigma_{i_{j}}}\right)}^{1/k}\right)\right)}^{1/q},{\left(1-\prod_{1\leq i_{1}<\ldots <i_{k}\leq n}\left(1-\prod_{j=1}^{k}{\left(1-{\left(1-{\left(\gamma_{i_{j}}^{U}\right)}^{q}\right)}^{n\sigma_{i_{j}}}\right)}^{1/k}\right)\right)}^{1/q}\right]\right\}\;\left\{\left[\prod_{1\leq i_{1}<\ldots <i_{k}\leq n}{\left(1-\prod_{j=1}^{k}{\left(1-{\left(\eta_{i_{j}}^{L}\right)}^{nq\sigma_{i_{j}}}\right)}^{1/k}\right)}^{1/q},\prod_{1\leq i_{1}<\ldots <i_{k}\leq n}{\left(1-\prod_{j=1}^{k}{\left(1-{\left(\eta_{i_{j}}^{U}\right)}^{nq\sigma_{i_{j}}}\right)}^{1/k}\right)}^{1/q}\right]\right\}\}\rangle .$$

Finally,

$$\frac{1}{C_{n}^{k}}\underset{1\leq i_{1}<\ldots <i_{k}\leq n}{⊕}{\left(\overset{k}{\underset{j=1}{⊗}}\left(n\sigma_{i_{j}}d_{i_{j}}\right)\right)}^{1/k}=\langle f^{*-1}\left(1-\prod_{1\leq i_{1}<\cdots <i_{k}\leq n}{\left(1-\prod_{j=1}^{k}{\left(1-{\left(1-f^{*}\left(\theta_{i_{j}}\right)\right)}^{n\sigma_{i_{j}}}\right)}^{1/k}\right)}^{\frac{1}{C_{n}^{k}}}\right),\cup_{r_{i_{j}}\in h_{i_{j}},\eta_{i_{j}}\in g_{i_{j}}}\;\{\left\{\left[{\left(1-\prod_{1\leq i_{1}<\ldots <i_{k}\leq n}{\left(1-\prod_{j=1}^{k}{\left(1-{\left(1-{\left(\gamma_{i_{j}}^{L}\right)}^{q}\right)}^{n\sigma_{i_{j}}}\right)}^{1/k}\right)}^{\frac{1}{C_{n}^{k}}}\right)}^{1/q},{\left(1-\prod_{1\leq i_{1}<\ldots <i_{k}\leq n}{\left(1-\prod_{j=1}^{k}{\left(1-{\left(1-{\left(\gamma_{i_{j}}^{U}\right)}^{q}\right)}^{n\sigma_{i_{j}}}\right)}^{1/k}\right)}^{\frac{1}{C_{n}^{k}}}\right)}^{1/q}\right]\right\}\;\left\{\left[\prod_{1\leq i_{1}<\ldots <i_{k}\leq n}{\left(1-\prod_{j=1}^{k}{\left(1-{\left(\eta_{i_{j}}^{L}\right)}^{nq\sigma_{i_{j}}}\right)}^{1/k}\right)}^{1/qC_{n}^{k}},\prod_{1\leq i_{1}<\ldots <i_{k}\leq n}{\left(1-\prod_{j=1}^{k}{\left(1-{\left(\eta_{i_{j}}^{U}\right)}^{nq\sigma_{i_{j}}}\right)}^{1/k}\right)}^{1/qC_{n}^{k}}\right]\right\}\}\rangle .$$

□

**Theorem 3.**  *Let* $d_{i}=\langle s_{\theta_{i}},\left(h_{i},g_{i}\right)\rangle \left(i=1,2,\ldots ,n\right)$ *be a collection of IVq-RDHLVs, if* $d_{i}=d=\langle s_{\theta },\left(h,g\right)\rangle$ *for all i, and there is only one MD and NMD in d, then*

(27) $$IVq-RDHLPHM^{\left(k\right)}\left(d_{1},d_{2},\ldots ,d_{n}\right)=d.$$

**Proof.**  According to $d_{i}=d=\langle s_{\theta },\left(h,g\right)\rangle$, we can have $Sup\left(d_{i},d_{j}\right)=1$ for all i,j=(1,2,3,…,n)(i≠j), then $\sigma_{i_{j}}=\frac{1}{n}$ hold for all *i*. According to Theorem 2, we obtain

$$IVq-RDHLPHM^{\left(k\right)}\left(d_{1},d_{2},\ldots ,d_{n}\right)=\langle f^{*-1}\left(1-\prod_{1\leq i_{1}<\cdots <i_{k}\leq n}{\left(1-\prod_{j=1}^{k}{\left(1-{\left(1-f^{*}\left(\theta_{i_{j}}\right)\right)}^{n\sigma_{i_{j}}}\right)}^{1/k}\right)}^{\frac{1}{C_{n}^{k}}}\right),\cup_{r_{i_{j}}\in h_{i_{j}},\eta_{i_{j}}\in g_{i_{j}}}\;\{\left\{\left[{\left(1-\prod_{1\leq i_{1}<\ldots <i_{k}\leq n}{\left(1-\prod_{j=1}^{k}{\left(1-{\left(1-{\left(\gamma_{i_{j}}^{L}\right)}^{q}\right)}^{n\sigma_{i_{j}}}\right)}^{1/k}\right)}^{\frac{1}{C_{n}^{k}}}\right)}^{1/q},{\left(1-\prod_{1\leq i_{1}<\ldots <i_{k}\leq n}{\left(1-\prod_{j=1}^{k}{\left(1-{\left(1-{\left(\gamma_{i_{j}}^{U}\right)}^{q}\right)}^{n\sigma_{i_{j}}}\right)}^{1/k}\right)}^{\frac{1}{C_{n}^{k}}}\right)}^{1/q}\right]\right\}\;\left\{\left[\prod_{1\leq i_{1}<\ldots <i_{k}\leq n}{\left(1-\prod_{j=1}^{k}{\left(1-{\left(\eta_{i_{j}}^{L}\right)}^{nq\sigma_{i_{j}}}\right)}^{1/k}\right)}^{1/qC_{n}^{k}},\prod_{1\leq i_{1}<\ldots <i_{k}\leq n}{\left(1-\prod_{j=1}^{k}{\left(1-{\left(\eta_{i_{j}}^{U}\right)}^{nq\sigma_{i_{j}}}\right)}^{1/k}\right)}^{1/qC_{n}^{k}}\right]\right\}\}\rangle \;=\langle f^{*-1}\left(1-\prod_{1\leq i_{1}<\cdots <i_{k}\leq n}{\left(1-\prod_{j=1}^{k}{\left(1-{\left(1-f^{*}\left(\theta_{i_{j}}\right)\right)}^{n\times \frac{1}{n}}\right)}^{1/k}\right)}^{\frac{1}{C_{n}^{k}}}\right),\cup_{r_{i_{j}}\in h_{i_{j}},\eta_{i_{j}}\in g_{i_{j}}}\;\{\left\{\left[{\left(1-\prod_{1\leq i_{1}<\ldots <i_{k}\leq n}{\left(1-\prod_{j=1}^{k}{\left(1-{\left(1-{\left(\gamma_{i_{j}}^{L}\right)}^{q}\right)}^{n\times \frac{1}{n}}\right)}^{1/k}\right)}^{\frac{1}{C_{n}^{k}}}\right)}^{1/q},{\left(1-\prod_{1\leq i_{1}<\ldots <i_{k}\leq n}{\left(1-\prod_{j=1}^{k}{\left(1-{\left(1-{\left(\gamma_{i_{j}}^{U}\right)}^{q}\right)}^{n\times \frac{1}{n}}\right)}^{1/k}\right)}^{\frac{1}{C_{n}^{k}}}\right)}^{1/q}\right]\right\}\;\left\{\left[\prod_{1\leq i_{1}<\ldots <i_{k}\leq n}{\left(1-\prod_{j=1}^{k}{\left(1-{\left(\eta_{i_{j}}^{L}\right)}^{nq\times \frac{1}{n}}\right)}^{1/k}\right)}^{1/qC_{n}^{k}},\prod_{1\leq i_{1}<\ldots <i_{k}\leq n}{\left(1-\prod_{j=1}^{k}{\left(1-{\left(\eta_{i_{j}}^{U}\right)}^{nq\times \frac{1}{n}}\right)}^{1/k}\right)}^{1/qC_{n}^{k}}\right]\right\}\}\rangle \;=\langle f^{*-1}\left(1-\prod_{1\leq i_{1}<\cdots <i_{k}\leq n}{\left(1-\prod_{j=1}^{k}{\left(f^{*}\left(\theta_{i_{j}}\right)\right)}^{1/k}\right)}^{\frac{1}{C_{n}^{k}}}\right),\cup_{r_{i_{j}}\in h_{i_{j}},\eta_{i_{j}}\in g_{i_{j}}}\;\{\left\{\left[{\left(1-\prod_{1\leq i_{1}<\ldots <i_{k}\leq n}{\left(1-\prod_{j=1}^{k}{\left({\left(\gamma_{i_{j}}^{L}\right)}^{q}\right)}^{1/k}\right)}^{\frac{1}{C_{n}^{k}}}\right)}^{1/q},{\left(1-\prod_{1\leq i_{1}<\ldots <i_{k}\leq n}{\left(1-\prod_{j=1}^{k}{\left({\left(\gamma_{i_{j}}^{U}\right)}^{q}\right)}^{1/k}\right)}^{\frac{1}{C_{n}^{k}}}\right)}^{1/q}\right]\right\}\;\left\{\left[\prod_{1\leq i_{1}<\ldots <i_{k}\leq n}{\left(1-\prod_{j=1}^{k}{\left(1-{\left(\eta_{i_{j}}^{L}\right)}^{q}\right)}^{1/k}\right)}^{1/qC_{n}^{k}},\prod_{1\leq i_{1}<\ldots <i_{k}\leq n}{\left(1-\prod_{j=1}^{k}{\left(1-{\left(\eta_{i_{j}}^{U}\right)}^{q}\right)}^{1/k}\right)}^{1/qC_{n}^{k}}\right]\right\}\}\rangle \;=\langle f^{*-1}\left(1-\prod_{1\leq i_{1}<\cdots <i_{k}\leq n}{\left(1-f^{*}\left(\theta_{i}\right)\right)}^{\frac{1}{C_{n}^{k}}}\right),\cup_{r_{i}\in h_{i},\eta_{i}\in g_{i}}\;\{\left\{\left[{\left(1-\prod_{1\leq i_{1}<\ldots <i_{k}\leq n}{\left(1-{\left(\gamma_{i}^{L}\right)}^{q}\right)}^{\frac{1}{C_{n}^{k}}}\right)}^{1/q},{\left(1-\prod_{1\leq i_{1}<\ldots <i_{k}\leq n}{\left(1-{\left(\gamma_{i}^{U}\right)}^{q}\right)}^{\frac{1}{C_{n}^{k}}}\right)}^{1/q}\right]\right\}\;\left\{\left[\prod_{1\leq i_{1}<\ldots <i_{k}\leq n}{\left(1-\left(1-{\left(\eta_{i}^{L}\right)}^{q}\right)\right)}^{1/qC_{n}^{k}},\prod_{1\leq i_{1}<\ldots <i_{k}\leq n}{\left(1-\left(1-{\left(\eta_{i}^{U}\right)}^{q}\right)\right)}^{1/qC_{n}^{k}}\right]\right\}\}\rangle \;=\langle f^{*-1}\left(1-{\left(1-f^{*}\left(\theta \right)\right)}^{\frac{1}{C_{n}^{k}}\times C_{n}^{k}}\right),\cup_{r\in h,\eta \in g}\;\{\left\{\left[{\left(1-{\left(1-{\left(\gamma^{l}\right)}^{q}\right)}^{\frac{1}{C_{n}^{k}}\times C_{n}^{k}}\right)}^{1/q},{\left(1-{\left(1-{\left(\gamma^{u}\right)}^{q}\right)}^{\frac{1}{C_{n}^{k}}\times C_{n}^{k}}\right)}^{1/q}\right]\right\}\;\left\{\left[{\left({\left(\eta^{l}\right)}^{q}\right)}^{C_{n}^{k}\times \frac{1}{qC_{n}^{k}}},{\left({\left(\eta^{u}\right)}^{q}\right)}^{C_{n}^{k}\times \frac{1}{qC_{n}^{k}}}\right]\right\}\}\rangle \;=\langle f^{*-1}\left(f^{*}\left(\theta \right)\right),\cup_{r\in h,\eta \in g},\left\{\left\{\left[{\left({\left(\gamma^{l}\right)}^{q}\right)}^{1/q},{\left({\left(\gamma^{u}\right)}^{q}\right)}^{1/q}\right]\right\},\left\{\left[\eta^{l},\eta^{u}\right]\right\}\right\}\rangle \;=\langle \theta ,\cup_{r\in h,\eta \in g},\left\{\left\{\left[\gamma^{l},\gamma^{u}\right]\right\},\left\{\left[\eta^{l},\eta^{u}\right]\right\}\right\}\rangle =d$$

□

**Theorem** **4.** *Let *$d_{i}=\langle s_{\theta_{i}},\left(h_{i},g_{i}\right)\rangle \left(i=1,2,\ldots ,n\right)$*be a collection of IVq-RDHLVs, if *$d^{-}=\min\left(d_{1},d_{2},\ldots ,d_{n}\right)$*and *$d^{+}=\max\left(d_{1},d_{2},\ldots ,d_{n}\right)$, *then*

(28) $$x\leq IVq-RDHLPHM^{\left(k\right)}\left(d_{1},d_{2},\ldots ,d_{n}\right)\leq y.$$

*where *$x=\frac{1}{C_{n}^{k}}\underset{1\leq i_{1}<\ldots <i_{k}\leq n}{⊕}{\left(\overset{k}{\underset{j=1}{⊗}}\left(n\sigma_{i_{j}}d^{-}\right)\right)}^{1/k}$*and *$y=\frac{1}{C_{n}^{k}}\underset{1\leq i_{1}<\ldots <i_{k}\leq n}{⊕}{\left(\overset{k}{\underset{j=1}{⊗}}\left(n\sigma_{i_{j}}d^{+}\right)\right)}^{1/k}$.

**Proof.**  From Definition 11, we can obtain

$$n\sigma_{i_{j}}d^{-}\leq n\sigma_{i_{j}}d_{i_{j}}\;\mathrm{and}\;\overset{k}{\underset{j=1}{⊗}}\left(n\sigma_{i_{j}}d^{-}\right)\leq \overset{k}{\underset{j=1}{⊗}}n\sigma_{i_{j}}d_{i_{j}}$$

Then,

$${\left(\overset{k}{\underset{j=1}{⊗}}\left(n\sigma_{i_{j}}d^{-}\right)\right)}^{1/k}\leq {\left(\overset{k}{\underset{j=1}{⊗}}n\sigma_{i_{j}}d_{i_{j}}\right)}^{1/k}$$

Further,

$$\underset{1\leq i_{1}<\ldots <i_{k}\leq n}{⊕}{\left(\overset{k}{\underset{j=1}{⊗}}\left(n\sigma_{i_{j}}d^{-}\right)\right)}^{1/k}\leq \underset{1\leq i_{1}<\ldots <i_{k}\leq n}{⊕}{\left(\overset{k}{\underset{j=1}{⊗}}n\sigma_{i_{j}}d_{i_{j}}\right)}^{1/k}$$

Thus,

$$\frac{1}{C_{n}^{k}}\underset{1\leq i_{1}<\ldots <i_{k}\leq n}{⊕}{\left(\overset{k}{\underset{j=1}{⊗}}\left(n\sigma_{i_{j}}d^{-}\right)\right)}^{1/k}\leq \frac{1}{C_{n}^{k}}\underset{1\leq i_{1}<\ldots <i_{k}\leq n}{⊕}{\left(\overset{k}{\underset{j=1}{⊗}}n\sigma_{i_{j}}d_{i_{j}}\right)}^{1/k}$$

which means that $x\leq IVq-RDHLPHM^{\left(k\right)}\left(d_{1},d_{2},\ldots ,d_{n}\right)$.

Similarly, we can also prove that $IVq-RDHLPHM^{\left(k\right)}\left(d_{1},d_{2},\ldots ,d_{n}\right)\leq y$. Thus, the proof of Theorem 4 is completed. □

Next, we will explore several special cases of the IVq-RDHLPHM operator when the parameter values changes.

**Case** **1.**  *When *k=1, *the proposed IVq-RDHLPHM operator is simplified to IVq-RDHL power average (IVq-RDHLPA) operator.*

(29) $$IVq-RDHLPHM^{\left(k\right)}\left(d_{1},d_{2},\ldots ,d_{n}\right)=\langle f^{*-1}\left(1-\prod_{1\leq i_{1}<\cdots <i_{k}\leq n}{\left(1-f^{*}\left(\theta_{i}\right)\right)}^{\sigma_{i}}\right),\cup_{r_{i}\in h_{i},\eta_{i}\in g_{i}}\;\left\{\left\{\left[{\left(1-\prod_{i=1}^{n}{\left(1-{\left(\gamma_{i}^{L}\right)}^{q}\right)}^{\sigma_{i}}\right)}^{1/q},{\left(1-\prod_{i=1}^{n}{\left(1-{\left(\gamma_{i}^{U}\right)}^{q}\right)}^{\sigma_{i}}\right)}^{1/q}\right]\right\},\left\{\left[\prod_{i=1}^{k}{\left(\eta_{i}^{L}\right)}^{\sigma_{i}},\prod_{i=1}^{k}{\left(\eta_{i}^{U}\right)}^{\sigma_{i}}\right]\right\}\right\}\rangle \;=\overset{n}{\underset{i=1}{⊕}}\sigma_{i}d_{i}=IVq-RDHLPA\left(d_{1},d_{2},\ldots ,d_{n}\right).$$

*Moreover, for all *$Sup\left(d_{i},d_{j}\right)=t>0,i\neq j$, *then the IVq-RDHLPHM operator is simplified to the IVq-RDHL average (IVq-RDHLA) operator.*

(30) $$IVq-RDHLPHM^{\left(k\right)}\left(d_{1},d_{2},\ldots ,d_{n}\right)=\langle f^{*-1}\left(1-\prod_{1\leq i_{1}<\cdots <i_{k}\leq n}{\left(1-f^{*}\left(\theta_{i}\right)\right)}^{1/n}\right),\cup_{r_{i}\in h_{i},\eta_{i}\in g_{i}}\;\left\{\left\{\left[{\left(1-\prod_{i=1}^{n}{\left(1-{\left(\gamma_{i}^{L}\right)}^{q}\right)}^{1/n}\right)}^{1/q},{\left(1-\prod_{i=1}^{n}{\left(1-{\left(\gamma_{i}^{U}\right)}^{q}\right)}^{1/n}\right)}^{1/q}\right]\right\},\left\{\left[\prod_{i=1}^{k}{\left(\eta_{i}^{L}\right)}^{1/n},\prod_{i=1}^{k}{\left(\eta_{i}^{U}\right)}^{1/n}\right]\right\}\right\}\rangle \;=\frac{1}{n}\overset{n}{\underset{i=1}{⊕}}d_{i}=IVq-RDHLA\left(d_{1},d_{2},\ldots ,d_{n}\right).$$

**Case** **2.**  *When *k=n, *the IVq-RDHLPHM operator is simplified to IVq-RDHL power geometric (IVq-RDHLPG) operator.*

(31) $$IVq-RDHLPHM^{\left(k\right)}\left(d_{1},d_{2},\ldots ,d_{n}\right)=\langle f^{*-1}\left(\prod_{j=1}^{k}{\left(1-{\left(1-f^{*}\left(\theta_{j}\right)\right)}^{n\sigma_{j}}\right)}^{\frac{1}{n}}\right),\cup_{r_{j}\in h_{j},\eta_{j}\in g_{j}}\;\left\{\left\{\left[\prod_{j=1}^{k}{\left(\gamma_{j}^{L}\right)}^{\sigma_{j}},\prod_{j=1}^{k}{\left(\gamma_{j}^{U}\right)}^{\sigma_{j}}\right]\right\},\left\{\left[{\left(1-\prod_{j=1}^{n}{\left(1-{\left(\eta_{j}^{L}\right)}^{q}\right)}^{\sigma_{j}}\right)}^{1/q},{\left(1-\prod_{j=1}^{n}{\left(1-{\left(\eta_{j}^{U}\right)}^{q}\right)}^{\sigma_{j}}\right)}^{1/q}\right]\right\}\right\}\rangle \;={\left(\overset{n}{\underset{j=1}{⊗}}n\sigma_{j}d_{j}\right)}^{1/n}=IVq-RDHLPG\left(d_{1},d_{2},\ldots ,d_{n}\right).$$

*Moreover, for all *$Sup\left(d_{i},d_{j}\right)=t>0,i\neq j$, *then the IVq-RDHLPHM operator is simplified to IVq-RDHL geometric (IVq-RDHLG) operator.*

(32) $$IVq-RDHLPHM^{\left(k\right)}\left(d_{1},d_{2},\ldots ,d_{n}\right)=\langle f^{*-1}\left(\prod_{j=1}^{k}{\left(f^{*}\left(\theta_{j}\right)\right)}^{\frac{1}{n}}\right),\cup_{r_{j}\in h_{j},\eta_{j}\in g_{j}}\;\left\{\left\{\left[\prod_{j=1}^{k}{\left(\gamma_{j}^{L}\right)}^{\frac{1}{n}},\prod_{j=1}^{k}{\left(\gamma_{j}^{U}\right)}^{\frac{1}{n}}\right]\right\},\left\{\left[{\left(1-\prod_{j=1}^{n}{\left(1-{\left(\eta_{j}^{L}\right)}^{q}\right)}^{\frac{1}{n}}\right)}^{1/q},{\left(1-\prod_{j=1}^{n}{\left(1-{\left(\eta_{j}^{U}\right)}^{q}\right)}^{\frac{1}{n}}\right)}^{1/q}\right]\right\}\right\}\rangle \;={\left(\overset{n}{\underset{j=1}{⊗}}d_{j}\right)}^{1/n}=IVq-RDHLG\left(d_{1},d_{2},\ldots ,d_{n}\right).$$

**Case** **3.**  *When*q=1*, the IVq-RDHLPHM operator is simplified to interval-valued dual hesitant linguistic PHM (IVDHLPHM) operator.*

(33) $$IVq-RDHLPHM^{\left(k\right)}\left(d_{1},d_{2},\ldots ,d_{n}\right)=\langle f^{*-1}\left(1-\prod_{1\leq i_{1}<\cdots <i_{k}\leq n}{\left(1-\prod_{j=1}^{k}{\left(1-{\left(1-f^{*}\left(\theta_{i_{j}}\right)\right)}^{n\sigma_{i_{j}}}\right)}^{1/k}\right)}^{\frac{1}{C_{n}^{k}}}\right),\cup_{r_{i_{j}}\in h_{i_{j}},\eta_{i_{j}}\in g_{i_{j}}}\;\{\left\{\left[1-\prod_{1\leq i_{1}<\ldots <i_{k}\leq n}{\left(1-\prod_{j=1}^{k}{\left(1-{\left(1-\left(\gamma_{i_{j}}^{L}\right)\right)}^{n\sigma_{i_{j}}}\right)}^{1/k}\right)}^{\frac{1}{C_{n}^{k}}},1-\prod_{1\leq i_{1}<\ldots <i_{k}\leq n}{\left(1-\prod_{j=1}^{k}{\left(1-{\left(1-\left(\gamma_{i_{j}}^{U}\right)\right)}^{n\sigma_{i_{j}}}\right)}^{1/k}\right)}^{\frac{1}{C_{n}^{k}}}\right]\right\}\;\left\{\left[\prod_{1\leq i_{1}<\ldots <i_{k}\leq n}{\left(1-\prod_{j=1}^{k}{\left(1-{\left(\eta_{i_{j}}^{L}\right)}^{n\sigma_{i_{j}}}\right)}^{1/k}\right)}^{1/C_{n}^{k}},\prod_{1\leq i_{1}<\ldots <i_{k}\leq n}{\left(1-\prod_{j=1}^{k}{\left(1-{\left(\eta_{i_{j}}^{U}\right)}^{n\sigma_{i_{j}}}\right)}^{1/k}\right)}^{1/C_{n}^{k}}\right]\right\}\}\rangle .$$

*Moreover, for all *$Sup\left(d_{i},d_{j}\right)=t>0,i\neq j$, *then the IVq-RDHLPHM operator is simplified to interval-valued dual hesitant linguistic HM (IVDHLHM) operator.*

(34) $$IVq-RDHLPHM^{\left(k\right)}\left(d_{1},d_{2},\ldots ,d_{n}\right)=\langle f^{*-1}\left(1-\prod_{1\leq i_{1}<\cdots <i_{k}\leq n}{\left(1-\prod_{j=1}^{k}{\left(f^{*}\left(\theta_{i_{j}}\right)\right)}^{1/k}\right)}^{\frac{1}{C_{n}^{k}}}\right),\cup_{r_{i_{j}}\in h_{i_{j}},\eta_{i_{j}}\in g_{i_{j}}}\;\{\left\{\left[1-\prod_{1\leq i_{1}<\ldots <i_{k}\leq n}{\left(1-\prod_{j=1}^{k}{\left(\gamma_{i_{j}}^{L}\right)}^{1/k}\right)}^{\frac{1}{C_{n}^{k}}},1-\prod_{1\leq i_{1}<\ldots <i_{k}\leq n}{\left(1-\prod_{j=1}^{k}{\left(\gamma_{i_{j}}^{U}\right)}^{1/k}\right)}^{\frac{1}{C_{n}^{k}}}\right]\right\}\;\left\{\left[\prod_{1\leq i_{1}<\ldots <i_{k}\leq n}{\left(1-\prod_{j=1}^{k}{\left(1-\left(\eta_{i_{j}}^{L}\right)\right)}^{1/k}\right)}^{1/C_{n}^{k}},\prod_{1\leq i_{1}<\ldots <i_{k}\leq n}{\left(1-\prod_{j=1}^{k}{\left(1-\left(\eta_{i_{j}}^{U}\right)\right)}^{1/k}\right)}^{1/C_{n}^{k}}\right]\right\}\}\rangle .$$

**Case** **4.**  *When* q=2 *, the IVq-RDHLPHM operator is simplified to interval-valued dual hesitant Pythagorean linguistic PHM (IVDHPLPHM) operator.*

(35) $$IVq-RDHLPHM^{\left(k\right)}\left(d_{1},d_{2},\ldots ,d_{n}\right)=\langle f^{*-1}\left(1-\prod_{1\leq i_{1}<\cdots <i_{k}\leq n}{\left(1-\prod_{j=1}^{k}{\left(1-{\left(1-f^{*}\left(\theta_{i_{j}}\right)\right)}^{n\sigma_{i_{j}}}\right)}^{1/k}\right)}^{\frac{1}{C_{n}^{k}}}\right),\cup_{r_{i_{j}}\in h_{i_{j}},\eta_{i_{j}}\in g_{i_{j}}}\;\{\left\{\left[{\left(1-\prod_{1\leq i_{1}<\ldots <i_{k}\leq n}{\left(1-\prod_{j=1}^{k}{\left(1-{\left(1-{\left(\gamma_{i_{j}}^{L}\right)}^{2}\right)}^{n\sigma_{i_{j}}}\right)}^{1/k}\right)}^{\frac{1}{C_{n}^{k}}}\right)}^{1/2},{\left(1-\prod_{1\leq i_{1}<\ldots <i_{k}\leq n}{\left(1-\prod_{j=1}^{k}{\left(1-{\left(1-{\left(\gamma_{i_{j}}^{U}\right)}^{2}\right)}^{n\sigma_{i_{j}}}\right)}^{1/k}\right)}^{\frac{1}{C_{n}^{k}}}\right)}^{1/2}\right]\right\}\;\left\{\left[\prod_{1\leq i_{1}<\ldots <i_{k}\leq n}{\left(1-\prod_{j=1}^{k}{\left(1-{\left(\eta_{i_{j}}^{L}\right)}^{2n\sigma_{i_{j}}}\right)}^{1/k}\right)}^{1/C_{n}^{k}},\prod_{1\leq i_{1}<\ldots <i_{k}\leq n}{\left(1-\prod_{j=1}^{k}{\left(1-{\left(\eta_{i_{j}}^{U}\right)}^{2n\sigma_{i_{j}}}\right)}^{1/k}\right)}^{1/C_{n}^{k}}\right]\right\}\}\rangle .$$

*In addition, for all* $Sup\left(d_{i},d_{j}\right)=t>0,i\neq j$ *, the IVq-RDHLPHM operator is simplified to interval-valued dual hesitant Pythagorean linguistic HM (IVDHPLHM) operator.*

(36) $$IVq-RDHLPHM^{\left(k\right)}\left(d_{1},d_{2},\ldots ,d_{n}\right)=\langle f^{*-1}\left(1-\prod_{1\leq i_{1}<\cdots <i_{k}\leq n}{\left(1-\prod_{j=1}^{k}{\left(f^{*}\left(\theta_{i_{j}}\right)\right)}^{1/k}\right)}^{\frac{1}{C_{n}^{k}}}\right),\cup_{r_{i_{j}}\in h_{i_{j}},\eta_{i_{j}}\in g_{i_{j}}}\;\{\left\{\left[{\left(1-\prod_{1\leq i_{1}<\ldots <i_{k}\leq n}{\left(1-\prod_{j=1}^{k}{\left(\gamma_{i_{j}}^{L}\right)}^{2/k}\right)}^{\frac{1}{C_{n}^{k}}}\right)}^{1/2},{\left(1-\prod_{1\leq i_{1}<\ldots <i_{k}\leq n}{\left(1-\prod_{j=1}^{k}{\left(\gamma_{i_{j}}^{U}\right)}^{2/k}\right)}^{\frac{1}{C_{n}^{k}}}\right)}^{1/2}\right]\right\}\;\left\{\left[\prod_{1\leq i_{1}<\ldots <i_{k}\leq n}{\left(1-\prod_{j=1}^{k}{\left(1-{\left(\eta_{i_{j}}^{L}\right)}^{2}\right)}^{1/k}\right)}^{1/2C_{n}^{k}},\prod_{1\leq i_{1}<\ldots <i_{k}\leq n}{\left(1-\prod_{j=1}^{k}{\left(1-{\left(\eta_{i_{j}}^{U}\right)}^{2}\right)}^{1/k}\right)}^{1/2C_{n}^{k}}\right]\right\}\}\rangle .$$

### 4.2. The Interval-Valued q-Rung Dual Hesitant Linguistic Power Weighted Hamy Mean (IVq-RDHLPWHM) Operator

*Let *$d_{i}\left(i=1,2,\ldots ,n\right)$*be a collection of IVq-RDHLVs and *k=1,2,…,n. *The corresponding weight vector can be denoted as*$w={\left(w_{1},w_{2},\ldots ,w_{n}\right)}^{T}$, *satisfying *$0\leq w_{i}\leq 1$*and *$\sum_{i=1}^{n}w_{i}=1$. *Then, the IVq-RDHLPWHM operator is expressed as*

(37) $$IVq-RDHLPWHM^{\left(k\right)}\left(d_{1},d_{2},\ldots ,d_{n}\right)=\frac{1}{C_{n}^{k}}\left(\underset{1\leq i_{1}<\cdots <i_{k}\leq n}{⊕}{\left(\overset{k}{\underset{j=1}{⊗}}\left(\frac{nw_{i_{j}}\left(1+T\left(d_{i_{j}}\right)\right)d_{i_{j}}}{\sum_{i=1}^{n}w_{i}\left(1+T\left(d_{i}\right)\right)}\right)\right)}^{1/k}\right),$$

*where *$C_{n}^{k}$*is the binomial coefficient, and *$\left(i_{1},i_{2},\ldots ,i_{k}\right)$*traverses all the k-tuple combination of *(1,2,…,n). *In addition, *$T\left(d_{i}\right)=\sum_{j=1,i\neq j}^{n}Sup\left(d_{i},d_{j}\right)$*and *$Sup\left(d_{i},d_{j}\right)$*represents the support for *$d_{i}$*from *$d_{j}$, *satisfying the conditions depicted in Definition 4.*

For convenience, we assume

(38) $$\delta_{i}=\frac{w_{i}\left(1+T\left(d_{i}\right)\right)}{\sum_{i=1}^{n}w_{i}\left(1+T\left(d_{i}\right)\right)},$$

then, Equation (37) can be transformed into

(39) $$IVq-RDHLPWHM^{\left(k\right)}\left(d_{1},d_{2},\ldots ,d_{n}\right)=\frac{1}{C_{n}^{k}}\left(\underset{1\leq i_{1}<\cdots <i_{k}\leq n}{⊕}{\left(\overset{k}{\underset{j=1}{⊗}}\left(n\delta_{i_{j}}d_{i_{j}}\right)\right)}^{1/k}\right),$$

where $0\leq \delta_{i}\leq 1$ and $\sum_{i=1}^{n}\delta_{i}=1$.

**Theorem** **5.** *Let* $d_{i}=\langle s_{\theta_{i}},\left(h_{i},g_{i}\right)\rangle \left(i=1,2,\ldots ,n\right)$ *be a collection of IVq-RDHLVs, then the results aggregated by the IVq-RDHLPWHM operator is also an IVq-RDHLV and *

(40) $$IVq-RDHLPWHM^{\left(k\right)}\left(d_{1},d_{2},\ldots ,d_{n}\right)=\langle f^{*-1}\left(1-\prod_{1\leq i_{1}<\cdots <i_{k}\leq n}{\left(1-\prod_{j=1}^{k}{\left(1-{\left(1-f^{*}\left(\theta_{i_{j}}\right)\right)}^{n\delta_{i_{j}}}\right)}^{1/k}\right)}^{\frac{1}{C_{n}^{k}}}\right),\;\cup_{r_{i_{j}}\in h_{i_{j}},\eta_{i_{j}}\in g_{i_{j}}}\{\left\{\left[{\left(1-\prod_{1\leq i_{1}<\ldots <i_{k}\leq n}{\left(1-\prod_{j=1}^{k}{\left(1-{\left(1-{\left(\gamma_{i_{j}}^{L}\right)}^{q}\right)}^{n\delta_{i_{j}}}\right)}^{1/k}\right)}^{\frac{1}{C_{n}^{k}}}\right)}^{1/q},{\left(1-\prod_{1\leq i_{1}<\ldots <i_{k}\leq n}{\left(1-\prod_{j=1}^{k}{\left(1-{\left(1-{\left(\gamma_{i_{j}}^{U}\right)}^{q}\right)}^{n\delta_{i_{j}}}\right)}^{1/k}\right)}^{\frac{1}{C_{n}^{k}}}\right)}^{1/q}\right]\right\}\;\left\{\left[\prod_{1\leq i_{1}<\ldots <i_{k}\leq n}{\left(1-\prod_{j=1}^{k}{\left(1-{\left(\eta_{i_{j}}^{L}\right)}^{nq\delta_{i_{j}}}\right)}^{1/k}\right)}^{1/qC_{n}^{k}},\prod_{1\leq i_{1}<\ldots <i_{k}\leq n}{\left(1-\prod_{j=1}^{k}{\left(1-{\left(\eta_{i_{j}}^{U}\right)}^{nq\delta_{i_{j}}}\right)}^{1/k}\right)}^{1/qC_{n}^{k}}\right]\right\}\}\rangle .$$

The specific proof process of Theorem 5 is omitted here because it is similar to the proof of Theorem 2.

**Theorem** **6.** *(Boundedness) Let *$d_{i}=\langle s_{\theta_{i}},\left(h_{i},g_{i}\right)\rangle \left(i=1,2,\ldots ,n\right)$*be a collection of IVq-RDHLVs, if *$d^{-}=\min\left(d_{1},d_{2},\ldots ,d_{n}\right)$*and *$d^{+}=\max\left(d_{1},d_{2},\ldots ,d_{n}\right)$, *then*

(41) $$x\leq IVq-RDHLPWHM^{\left(k\right)}\left(d_{1},d_{2},\ldots ,d_{n}\right)\leq y.$$

*where *$x=\frac{1}{C_{n}^{k}}\underset{1\leq i_{1}<\ldots <i_{k}\leq n}{⊕}{\left(\overset{k}{\underset{j=1}{⊗}}\left(n\delta_{i_{j}}d^{-}\right)\right)}^{1/k}$*and *$y=\frac{1}{C_{n}^{k}}\underset{1\leq i_{1}<\ldots <i_{k}\leq n}{⊕}{\left(\overset{k}{\underset{j=1}{⊗}}\left(n\delta_{i_{j}}d^{+}\right)\right)}^{1/k}$.

**Proof.**  From Definition 11, we can obtain

$$n\delta_{i_{j}}d^{-}\leq n\delta_{i_{j}}d_{i_{j}}\mathrm{and}\overset{k}{\underset{j=1}{⊗}}\left(n\delta_{i_{j}}d^{-}\right)\leq \overset{k}{\underset{j=1}{⊗}}n\delta_{i_{j}}d_{i_{j}}$$

Then,

$${\left(\overset{k}{\underset{j=1}{⊗}}\left(n\delta_{i_{j}}d^{-}\right)\right)}^{1/k}\leq {\left(\overset{k}{\underset{j=1}{⊗}}n\delta_{i_{j}}d_{i_{j}}\right)}^{1/k}$$

Further,

$$\underset{1\leq i_{1}<\ldots <i_{k}\leq n}{⊕}{\left(\overset{k}{\underset{j=1}{⊗}}\left(n\delta_{i_{j}}d^{-}\right)\right)}^{1/k}\leq \underset{1\leq i_{1}<\ldots <i_{k}\leq n}{⊕}{\left(\overset{k}{\underset{j=1}{⊗}}n\delta_{i_{j}}d_{i_{j}}\right)}^{1/k}$$

Thus,

$$\frac{1}{C_{n}^{k}}\underset{1\leq i_{1}<\ldots <i_{k}\leq n}{⊕}{\left(\overset{k}{\underset{j=1}{⊗}}\left(n\delta_{i_{j}}d^{-}\right)\right)}^{1/k}\leq \frac{1}{C_{n}^{k}}\underset{1\leq i_{1}<\ldots <i_{k}\leq n}{⊕}{\left(\overset{k}{\underset{j=1}{⊗}}n\delta_{i_{j}}d_{i_{j}}\right)}^{1/k}$$

which means that $x\leq IVq-RDHLPWHM^{\left(k\right)}\left(d_{1},d_{2},\ldots ,d_{n}\right)$.□

Similarly, we can also prove that $IVq-RDHLPWHM^{\left(k\right)}\left(d_{1},d_{2},\ldots ,d_{n}\right)\leq y$. Thus, the proof of Theorem 6 is completed.

## 5. A MADM Method under IVq-RDHLSs

For a MADM problem, DMs express their assessment with IVq-RDHLSs information. Assume that there are *m* alternatives $\left\{A_{1},A_{2},\ldots ,A_{m}\right\}$ that will be evaluated, and *n* attributes $\left\{C_{1},C_{2},\ldots ,C_{n}\right\}$ should be considered in the process of decision making. The weight vector of the attributes is $w={\left(w_{1},w_{2},\ldots ,w_{n}\right)}^{T}$, which satisfy that $0\leq w_{j}\leq 1$ and $\sum_{j=1}^{n}w_{j}=1$. When evaluating, DMs use IVq-RDHLS $d_{ij}=\langle s_{\theta_{ij}},\left(h_{ij},g_{ij}\right)\rangle \left(i=1,2,\ldots ,m;j=1,2,\ldots ,n\right)$ to express their evaluation of the attribute $C_{j}\left(j=1,2,\ldots ,n\right)$ of $A_{i}\left(i=1,2,\ldots ,m\right)$. Later, the overall evaluation information can be collected, and a decision matrix can be obtained, which can be written as $R_{ij}={\left(d_{ij}\right)}_{m\times n}$. In the following, we introduce the steps of how to determine the ranks of alternatives based on the IVq-RDHLPWHM operator.

Step 1. Standardize the original decision values. Before aggregation, the original decision values should be standardized according to the following formula:

(42) $$d_{ij}=\left\{ \begin{array}{l} \langle s_{\theta_{ij}},\left(h_{ij},g_{ij}\right)\rangle C_{j}\;is\;benefit\;type\\ \langle s_{\theta_{ij}},\left(g_{ij}h_{ij}\right)\rangle C_{j}\;is\;cost\;type \end{array}\right.,$$

Step 2. Calculate the support

(43) $$Sup\left(d_{il},d_{im}\right)=1-d\left(d_{il},d_{im}\right),\left(l,m=1,2,\ldots ,n,l\neq m\right),$$

Step 3. Compute the $T\left(d_{ij}\right)$ between the two IVq-RDHLS $d_{il}$ and $d_{im}$ by

(44) $$T\left(d_{ij}\right)=\sum_{l,m=1,l\neq m}^{n}Sup\left(d_{il},d_{im}\right).$$

Step 4. Compute the power weight $\delta_{ij}$ associated with IVq-RDHLSs by

(45) $$\delta_{ij}=\frac{w_{i}\left(1+T\left(d_{ij}\right)\right)}{\sum_{i=1}^{n}w_{i}\left(1+T\left(d_{ij}\right)\right)}.$$

Step 5. Calculate the overall evaluation values $d_{i}$ of the alternative $A_{i}$ by

(46) $$d_{i}=IVq-RDHLPWHM^{\left(k\right)}\left(d_{i1},d_{i2},\ldots ,d_{in}\right)$$

Step 6. Calculate the score values of $d_{i}\left(i=1,2,\ldots ,n\right)$ according to the Equation (20).

Step 7. Rank all the alternatives according to the score values, and choose the best alternative.

## 6. A Case Study in Assessment Indicator System of Patient Admission Evaluation

With the development of society, the aging of the population and the improvement of health awareness, people’s medical needs are increasing rapidly. However, medical resources, such as bed resources, medical technology, and operating rooms, are limited. When scarce resources cannot accommodate a large number of hospitalized patients, a feasible solution is to prioritize the patient’s hospitalization list. In practical MADM problems, there are always qualitative criteria whose values can hardly be depicted by crisp values, such as the level of pain, the severity of illness, etc. The IVq-RDHLs provide a new and powerful technique to represent the qualitative judgments of experts. Therefore, in order to assess the relative priorities of patients for treatment on a waiting list, we construct an evaluation index system to allow the patients with a high degree of disease severity to be hospitalized.

### 6.1. Patient Admission Evaluation Criteria

In this section, we build a patient admission evaluation index system for general patient prioritization. In practical problems, the evaluation of patient prioritization involves multiple factors and multiple indicators, such as the level of pain, the severity of illness, and the impact on the patient’s life year, etc. To solve this problem, Li et al. [47] conducted an investigation on the admission process and obtained four dimensions, including clinical and functional disorders (*C*_1_), expected outcomes (*C*_2_), social factors (*C*_3_), patient’s basic information (*C*4), as shown in Table 1.

**Example** **3.** *Suppose that there are four patients *$A_{i}\left(i=1,2,3,4\right)$*needed to be considered for patient admission. The parameters *$C_{j}\left(j=1,2,3,4\right)$*are employed in assessing the patients, in which *$C_{1}$*represents clinical and functional disorders, *$C_{2}$*represents expected outcomes, *$C_{3}$*represents social factors, and *$C_{4}$*represents patient basic information, whose weight vector is *$w={\left(0.3,0.3,0.2,0.2\right)}^{T}$*. Doctors mainly express their assessment information on patients in 5 levels {s_1_ = noting, s_2_=low, s_3_ = medium, s_4_ = high, s_5_ = very high}. To express the evaluation information of the patient’s symptoms in detail, doctors request to evaluate the four patients *$A_{i}\left(i=1,2,3,4\right)$*from the perspective of the four parameters *$C_{j}(j=1,2,3,4)$*by IVq-RDHLs. Then, a decision matrix *$d_{ij}=\langle s_{\theta_{ij}},\left(h_{ij},g_{ij}\right)\rangle$(i=1,2,3,4;j=1,2,3,4)*consisting of IVq-RDHLs can be obtained, which is shown in*Table 2.

Step 1. After analysis, it is obvious that all attributes belong to benefit type, so there is no need to standardize the original matrix according to Equation (42).

Step 2. Calculate the support $Sup\left(d_{il},d_{im}\right)$ according to Equation (43) (suppose we choose LSF1 in the calculation process). For convenience, we use $S^{lm}$ to represent the value $Sup\left(d_{il},d_{im}\right)$(l,m=1,2,3,4;i=1,2,3,4;l≠m). After calculation,

$$S^{12}=S^{21}=\left(0.9961,0.9871,0.9953,0.9957\right);\;S^{13}=S^{31}=\left(0.9937,0.9913,0.9932,0.9826\right);\;S^{14}=S^{41}=\left(0.9894,0.9779,0.9668,0.9705\right)\;S^{23}=S^{32}=\left(0.9828,0.9911,0.9902,0.9738\right).\;S^{24}=S^{42}=\left(0.9851,0.9860,0.9774,0.9896\right)\;S^{34}=S^{43}=\left(0.9937,0.9907,0.9647,0.9820\right)$$

Step 3. Calculate $T\left(d_{ij}\right)$ according to Equation (44). The symbol *T* represents the value $T\left(d_{ij}\right)$,

$$T=\left[\begin{array}{l} 2.9792\;2.9640\;2.9702\;2.9682\\ 2.9563\;2.9642\;2.9731\;2.9545\\ 2.9553\;2.9629\;2.9481\;2.9089\\ 2.9488\;2.9591\;2.9384\;2.9421 \end{array}\right]$$

Step 4. Calculate the power weight $\delta_{ij}$ according to Equation (45), and we have

$$\sigma_{ij}=\left[\begin{array}{l} 0.3006\;0.2995\;0.2000\;0.1999\\ 0.2996\;0.3002\;0.2006\;0.1996\\ 0.3006\;0.3012\;0.2001\;0.1981\\ 0.3000\;0.3008\;0.1995\;0.1997 \end{array}\right]$$

Step 5. For the patients $A_{i}\left(i=1,2,3,4\right)$, utilize the IVq-RDHLPWHM operator to calculate the evaluation $d_{i}\left(i=1,2,3,4\right)$. (Assume that *k* = 2 and *q* = 4.) We omit them here because the aggregation results are complicated.

Step 6. Calculate the score values $S\left(d_{i}\right)\left(i=1,2,3,4\right)$ of the overall evaluation values, and we have

$$S\left(d_{1}\right)=0.0424;\;S\left(d_{2}\right)=0.0745;\;S\left(d_{3}\right)=0.0354;\;S\left(d_{4}\right)=0.0211$$

Step 7. According to the score values $S(d_{i})(i=1,2,3,4)$, the ranking orders of the patients can be determined, that is $A_{2}>A_{1}>A_{3}>A_{4}$, which indicates that $A_{2}$ is the optimal patient that should be admitted to the hospital.

### 6.2. Sensitivity Analysis

From Definition 12, we can find that the parameters *k*, *q,* and LSF *f* play an important role in calculating the final decision results. Therefore, it is of high necessity to explore the influence on the final score values and ranking orders of alternatives. In this section, we explore how the parameters *k*, *q* and LSF affect the outcome of the decision, respectively.

#### 6.2.1. The Impact of the Parameter *q*

In this part, we explore the influence of the parameter *q* on the final results. In order to do that, we select different values of *q* to calculate the results, and the results are shown in Table 3. For convince, we assume *k* = 2 and choose the LSF 1 when aggregating. The data in Table 3 tell us that the score values increase as the value of *q* increases. Although the score values are different, the ranking orders are the same $A_{2}>A_{1}>A_{3}>A_{4}$, and $A_{2}$ is the optimal patient that should be admitted. Then, how to determine the appropriate value of *q* is a meaningful and worthy issue. Considering this, the method proposed by Feng et al. [3] suggests that the value of *q* should be the smallest integer that makes $0\leq {\left({\left(\gamma^{U}\right)}^{+}\right)}^{q}+{\left({\left(\eta^{U}\right)}^{+}\right)}^{q}\leq 1\left(q\geq 1\right)$. For example, an evaluation value produced by a DM using an IVq-RDHLE is $\langle s_{3},\left\{\left\{\left[0.5,0.6\right],\left[0.6,0.9\right]\right\},\left\{\left[0.2,0.5\right],\left[0.4,0.7\right]\right\}\right\}\rangle$. As $0.9^{3}+0.7^{3}=$ 1.072>1 and $0.9^{4}+0.7^{4}=0.8962<1$, then the value of *q* should be taken as 4.

#### 6.2.2. The Influence of the Parameter *k*

In this part, we discuss the impact of parameter *k* on the IVq-RDHLPWHM operator. Then, we assume the parameters *q* and LSF are stable and change the values of *k*. Based on this idea, we calculate the final results using the IVq-RDHLPWHM operator and obtain the results, as shown in Table 4. From Table 4, the score values of the four patients are obviously different. Specifically, the score values decrease when the parameter *k* increases. However, the ranking orders have not changed, i.e., $A_{2}>A_{1}>A_{3}>A_{4}$. In practical problems, the appreciated value of *k* can be selected according to the preference of the DM. When the DM has a positive attitude towards the alternatives, a smaller value of *k* will be selected.

#### 6.2.3. The Influence of the LSF

Obviously, LSF *f* have an important impact on the final results. Therefore, we select different LSF *f* values to solve Example 3, and the score values are shown in Table 5. It is found from Table 5 that with different *f* values, the score values of the alternatives are different. However, $A_{2}>A_{1}>A_{3}>A_{4}$ is always the ranking orders of alternative, and $A_{2}$ is the optimal alternative. Different LSF *f* values represent the understanding of a semantic gap by different DMs. In reality, the appropriate LSF *f* can be selected according to actual needs. If the DM thinks that adjacent semantics are equal, we can choose LSF1. If the DM has a positive attitude towards semantics, choose LSF2; otherwise, choose LSF3. All in all, the existence of LSF proves the practicality and flexibility of the new method.

### 6.3. Validity Analysis

In this section, we perform a comparative analysis between the two existing methods and our method. Later, we divided the comparison into two subsections as follows.

#### 6.3.1. Compared with the Method Based on the IVq-RDHLWMSM Operator

In this subsection, the method based on the IVq-RDHLWMSM operator proposed by Feng et al. [35] and the method we proposed are used to calculate the decision results, and the results are shown in Table 6. In addition, we choose different values of *k* when using the IVq-RDHLWMSM operator [35] and choose different LSF values when using the IVq-RDHLPWHM operator. Although the score values are obtained by different methods and different parameters, the ranking orders are the same. To be precise, compared with the method proposed by Feng et al. [35], our method can consider the understanding of different semantics according to the adjustment of LSF. Therefore, our method has strong flexibility compared to the IVq-RDHLWMSM operator proposed by Feng et al.’s [35].

#### 6.3.2. Compared with Du et al.’s Method

**Example** **4.** *(Revised from Du et al. [48]) A hospital intends to choose a supplier of medical equipment, and the choice of the supplier is affected by many factors. Suppose that there are four suppliers* $A_{i}\left(i=1,2,3,4\right)$ *that should be evaluated based on the four attributes* $C_{j}\left(j=1,2,3,4,5\right)$ *: the quality (* $C_{1}$ *), the price (* $C_{2}$ *), the service performance (* $C_{3}$ *), and the user evaluation (* $C_{4}$ *). The weight vector of the attributes is* $w={\left(0.29,0.28,0.18,0.25\right)}^{T}$ *. When evaluating, DMs were advised to use the interval-valued Pythagorean fuzzy linguistic variables (IVPFLVs) to express their opinion. Then, the decision matrix composed of IVPFLVs is omitted here, and it can be found in reference [48]. Later, two methods based on the IVPFLWA operator [48] and the IVq-RDHLPWHM operator were conducted to calculate the final ranking results. The results presented in Table 7 depict that the score values of each alternative are different, but the ranking orders are the same. In other words, our method cannot only deal with the problems under the IVq-RDHLVs environment but also solve the problems under the IVPFLVs fuzzy environment, which shows the usefulness and power of our proposed method.*

### 6.4. Advantages of the Proposed Method

In this section, we analyze the advantages and strengths of our proposed method point by point.

#### 6.4.1. The Flexibility of Its Operation

In most real MADM problems, DMs may have different semantic preference. The LSF is well-known for its ability to match different DMs’ semantic translation requirements. Our method based on IVq-RDHLPWHM also allows the DMs to choose the appropriate LSFs according to personal preference and the actual semantic environment for selecting the most suitable alternative. To illustrate this advantage, we use our method to solve the problem of Example 4 and obtain the final results by different LSFs (see Table 8). It can be clearly seen from Table 8 that although the score values obtained by different LSFs are different, the ranking orders calculated by different LSFs are completely the same. Therefore, our method can consider more semantic gaps and has more flexibility in solving MADM problems.

#### 6.4.2. Its Capability of Effectively Dealing with DMs’ Unreasonable Evaluation Values

The IVq-RDHLPWHM operator we proposed combined the PA operator and Hamy mean operator. The PA operator is famous for its ability to weaken the influence of extreme evaluation values on the final result because it considers the power weighting of attributes. In the decision-making process, influenced by knowledge background and educational experience, DMs may feel hesitant or have a prejudice against some alternatives; as a result, they may give egregiously high or low arguments when providing assessment information. Therefore, our method can eliminate the influence of extreme values and make the decision results more reasonable.

#### 6.4.3. It Powerfully Deals with the Complex Interrelationship among Multiple Attributes When Aggregating

The IVq-RDHLPWHM operator combines the PA operator and the HM operator. The HM operator can handle the complex interrelationships among attributes. Further, we use the IVq-RDHLPWHM operator to solve Example 4 by using different values of *k* and obtain the final results (presented in Table 9). Specifically, the value of parameter *k* indicates how to consider the relationship among attributes in the calculation process. However, the final ranking result of the alternatives shows that no matter what the values of *k*, the ranking orders are the same. Therefore, our method is robust to deal with the MADM problems because it can flexibly handle the correlation among attributes.

#### 6.4.4. It Effectively Expresses DM’s Evaluation Comprehensively

**Example** **5.** *In Example 4, DMs express their assessment of alternatives using the IVPFLs. In order to illustrate the advantage, we change the assessment of attribute* $C_{3}$ *of* $A_{3}$ *with* $\left\{s_{5},\left\{\left[0.8,0.9\right]\right\},\left\{\left[0.4,0.5\right]\right\}\right\}$ *. Then, use the IVPFLWA operator [48] and our method to solve the problem and obtain the final results (shown in Table 10). Obviously, we can find that the IVPFLWA operator cannot deal with the problem, but our method can still obtain the results. Next, we analyze the reasons for this result. The IVPFLWA operator can only solve the problem that satisfies that the sum of the second power of MG and the second power of NMG should be smaller than or equal to one. However, our method can break the constraint according to the definition of the IVq-RDHLPWHM operator. To be more precise,* $0.9^{2}+0.5^{2}=1.06>1$ *, but if we set* q=3 *, then* $0.9^{3}+0.5^{3}=0.854<1$ *. Therefore, the IVq-RDHLPWHM operator can still deal with Example 5 and obtain the ranking orders, i.e.,* $A_{3}>A_{4}>A_{2}>A_{1}$ *, but the IVPFLWA operator cannot solve it. The parameter of q makes DMs describe more information compared with the IVPFLs, which provide a useful tool for DMs when expressing their evaluation on the alternatives more comprehensively.*

### 6.5. Summarization

Based on the above analysis, we have summarized the characteristics of the existing MADM and displayed them in Table 11. From Table 11, it is obvious that our proposed method has more advantages in solving MADM problems compared with other methods. Next, we mainly analyze the following three aspects: (1) Compared with the method based on the IVq-RDHLWMSM operator proposed by Feng et al. [35], IVq-RDHLPWHM can effectively handle DMs’ extreme evaluation values; thereby, the decision results are more accurate and realistic. (2) In addition, the operational laws of our method are more flexible than those of the IVq-RDHLWMSM operator [35] because of the existence of LSF. By choosing different LSFs, the IVq-RDHLPWHM operator can capture the subjective evaluation of DMs, and it can make the final decision results more valuable. For example, a DM thinks that the semantic gap between “extremely bad” and “very bad” is smaller than “very good” and “extremely good”. When aggregating the evaluation values, we should choose LSF 2 to make the final decision results more reasonable. (3) Compared with the method based on the IVPFLWA operator [48], IVq-RDHLPWHM can capture the complicated relationship among the attributes and use it in the calculation of the final results. In addition, the applications of the IVq-RDHLPWHM operator are wider as it can deal with a larger information space, which shows that our method can effectively reduce the loss of evaluation information. In the condition of the DMs believe that adjacent semantics are not equal, the proposed IVq-RDHLPWHM operator based on LSF in this article can handle this issue well. Moreover, the IVq-RDHLPWHM operator can also handle the MADM problem, which is under the condition of the complex interrelationship among attributes and the extreme values proposed by DMs. All in all, our method is more robust and effective than existing methods when dealing with MADM problems.

## 7. Conclusions

In this paper, we introduce a new MADM method under the IVq-RDHLs fuzzy environment. Firstly, we proposed new operational rules of IVq-RDHLs that can satisfy different DMs’ semantic translation requirements. Secondly, inspired by the idea of the PHM operator and IVq-RDHLs, the IVq-RDHLPHM operator and the IVq-RDHLPWHM operator were proposed, which can not only reduce the negative influence of extreme values but also consider the interrelationships among attributes. Thirdly, we put forward a method based on the IVq-RDHLPWHM operator and show the main steps to dealing with MADM problems that involve IVq-RDHLs fuzzy information. Finally, numerical examples were used to reveal the validity of our method, and comparative analyses were used to explain the powerfulness of our methods. After the comparative analysis, it is obvious that the proposal of the IVq-RDHLPHM operator and the IVq-RDHLPWHM operator provide new solutions for MADM problems. It can not only deal with the understanding of different semantics between LTs by DMs but also capture the relationship among attributes and eliminate the influence of extreme values on the final decision. In the future, we will extend the decision-making methods to more actual MADM problems. In addition, combining IVq-RDHLs with traditional multi-objective decision-making methods (such as TOPSIS, AHP, etc.) to propose novel and powerful MADM methods is a good research direction. Moreover, we will explore more methods for IVq-RDHL information and apply them in modern realistic decision-making situations.

## Figures and Tables

**Table 1:** The evaluation criteria of patient admission.

Parameters	Brief Description
Clinical and functional disorders (*C*_1_).	Clinical and functional disorders are an essential dimension of the indicator system, which describe the severities of patients’ diseases and the degrees of treatment needed in terms of the disease characteristics. It includes disease severity, pain level, etc.
Expected outcomes (*C*_2_)	Expected outcomes refer to the effectiveness of treatment after hospitalization from the hospital’s point of view. To be precise, before admission, the hospital has the right to evaluate if the patients have expected negative effects after receiving treatment, such as mortality. In this sense, it includes the difficulty of treatment, the complication probability, etc.
Social factors (*C*_3_)	When considering the admission of patients, we need to maximize social welfare from a moral point of view. In this regard, social factors include resource consumption during waiting periods, limitations in doing activities of daily living and so on.
Patient basic information (*C*_4_)	The basic information of the patient should be considered in the comprehensive assessment process. For example, when other conditions are the same, patients who wait longer will be given higher priorities for treatment. A patient’s basic information can be described as follows: gender, age, waiting time under the same condition, etc.

**Table 2:** The interval-valued q-rung dual hesitant linguistic decision matrix D.

	*A*_1_	*A*_2_
*C*_1_	$\langle s_{2},\left\{\left\{\left[0.6,0.7\right],\left[0.5,0.8\right]\right\},\left\{\left[0.3,0.4\right],\left[0.4,0.5\right]\right\}\right\}\rangle$	$\langle s_{2},\left\{\left\{\left[0.7,0.8\right],\left[0.8,0.9\right]\right\},\left\{\left[0.2,0.3\right],\left[0.2,0.5\right]\right\}\right\}\rangle$
*C*_2_	$\langle s_{1},\left\{\left\{\left[0.6,0.7\right]\right\},\left\{\left[0.1,0.4\right],\left[0.3,0.5\right]\right\}\right\}\rangle$	$\langle s_{3}\left\{\left[0.4,0.6\right],\left[0.6,0.7\right]\right\},\left\{\left[0.1,0.3\right],\left[0.3,0.4\right]\right\}\rangle$
*C*_3_	$\langle s_{3},\left\{\left\{\left[0.5,0.6\right],\left[0.6,0.9\right]\right\},\left\{\left[0.2,0.5\right],\left[0.4,0.6\right]\right\}\right\}\rangle$	$\langle s_{4},\left\{\left[0.6,0.8\right]\right\},\left\{\left[0.2,0.4\right],\left[0.3,0.4\right]\right\}\rangle$
*C*_4_	$\langle s_{4},\left\{\left\{\left[0.4,0.6\right],\left[0.7,0.8\right]\right\},\left\{\left[0.1,0.2\right]\right\}\right\}\rangle$	$\langle s_{5},\left\{\left[0.5,0.6\right],\left[0.7,0.9\right]\right\},\left\{\left[0.1,0.2\right],\left[0.3,0.6\right]\right\}\rangle$
	*A*_3_	*A*_4_
*C*_1_	$\langle s_{2},\left\{\left\{\left[0.5,0.6\right]\right\},\left\{\left[0.5,0.7\right],\left[0.6,0.7\right]\right\}\right\}\rangle$	$\langle s_{3},\left\{\left\{\left[0.3,0.6\right],\left[0.5,0.8\right]\right\},\left\{\left[0.4,0.6\right],\left[0.5,0.6\right]\right\}\right\}\rangle$
*C*_2_	$\langle s_{3},\left\{\left\{\left[0.4,0.5\right],\left[0.5,0.6\right]\right\},\left\{\left[0.4,0.5\right],\left[0.5,.0.7\right]\right\}\right\}\rangle$	$\langle s_{2},\left\{\left\{\left[0.3,0.5\right],\left[0.5,0.7\right]\right\},\left\{\left[0.4,0.6\right],\left[0.6,0.7\right]\right\}\right\}\rangle$
*C*_3_	$\langle s_{1},\left\{\left\{\left[0.4,0.6\right],\left[0.6,0.7\right]\right\},\left\{\left[0.2,0.4\right],\left[0.3,0.6\right]\right\}\right\}\rangle$	$\langle s_{4},\left\{\left\{\left[0.3,0.5\right]\right\},\left\{\left[0.6,0.8\right],\left[0.8,0.9\right]\right\}\right\}\rangle$
*C*_4_	$\langle s_{5},\left\{\left\{\left[0.6,0.8\right],\left[0.7,0.8\right]\right\},\left\{\left[0.4,0.6\right],\left[0.5,0.6\right]\right\}\right\}\rangle$	$\langle s_{1},\left\{\left\{\left[0.1,0.3\right],\left[0.2,0.4\right]\right\},\left\{\left[0.6,0.8\right],\left[0.7,0.8\right]\right\}\right\}\rangle$

**Table 3:** Score values of alternatives $A_{i}\left(i=1,2,3,4\right)$ when q∈[1,5] based on IVq-RDHLPWHM operator (*k* = 2).

*q*	$\mathrm{Score}\;\mathrm{Values}\;S\left(d_{i}\right)\left(i=1,2,3,4\right)$	Ranking Orders
*q* = 1	$S\left(d_{1}\right)=0.0281$; $S\left(d_{2}\right)=0.0508$; $S\left(d_{3}\right)=0.0241$; $S\left(d_{4}\right)=0.0139$	$A_{2}>A_{1}>A_{3}>A_{4}$
*q* = 2	$S\left(d_{1}\right)=0.0330;S\left(d_{2}\right)=0.0594$; $S\left(d_{3}\right)=0.0263$; $S\left(d_{4}\right)=0.0145$	$A_{2}>A_{1}>A_{3}>A_{4}$
*q* = 3	$S\left(d_{1}\right)=0.0381$; $S\left(d_{2}\right)=0.0677$; $S\left(d_{3}\right)=0.0307$; $S\left(d_{4}\right)=0.0176$	$A_{2}>A_{1}>A_{3}>A_{4}$
*q* = 4	$S\left(d_{1}\right)=0.0424$; $S\left(d_{2}\right)=0.0745$; $S\left(d_{3}\right)=0.0354$; $S\left(d_{4}\right)=0.0211$	$A_{2}>A_{1}>A_{3}>A_{4}$
*q* = 5	$S\left(d_{1}\right)=0.0458$; $S\left(d_{2}\right)=0.0797$; $S\left(d_{3}\right)=0.0398$; $S\left(d_{4}\right)=0.0246$	$A_{2}>A_{1}>A_{3}>A_{4}$

**Table 4:** Score values and ranking results of Example 3 with different values of *k* in the IVq-RDHLPWHM operator (*q* = 4).

*k*	$\mathrm{Score}\;\mathrm{Values}\;S\left(d_{i}\right)\left(i=1,2,3,4\right)$	Ranking Orders
*k* = 1	$S\left(d_{1}\right)=0.0480$ $;\;S\left(d_{2}\right)=0.0827$ $;\;S\left(d_{3}\right)=0.0440$ $;\;S\left(d_{4}\right)=0.0276$	$A_{2}>A_{1}>A_{3}>A_{4}$
*k* = 2	$S\left(d_{1}\right)=0.0424$ $;\;S\left(d_{2}\right)=0.0745$ $;\;S\left(d_{3}\right)=0.0354$ $;\;S\left(d_{4}\right)=0.0211$	$A_{2}>A_{1}>A_{3}>A_{4}$
*k* = 3	$S\left(d_{1}\right)=0.0404$ $;\;S\left(d_{2}\right)=0.0717$ $;\;S\left(d_{3}\right)=0.0323$ $;\;S\left(d_{4}\right)=0.0188$	$A_{2}>A_{1}>A_{3}>A_{4}$
*k* = 4	$S\left(d_{1}\right)=0.2044$ $;\;S\left(d_{2}\right)=0.2408$ $;\;S\left(d_{3}\right)=0.1821$ $;\;S\left(d_{4}\right)=0.0927$	$A_{2}>A_{1}>A_{3}>A_{4}$

**Table 5:** Score functions and ranking orders by different LSFs (*q* = 4, *k* = 2).

Parameters	$\mathrm{Score}\;\mathrm{Values}\;S\left(d_{i}\right)\left(i=1,2,3,4\right)$	Ranking Orders
Our method based on LSF1 (*t* = 3)	$S\left(d_{1}\right)=0.0424$$;\;S\left(d_{2}\right)=0.0745$; $S\left(d_{3}\right)=0.0354$$;\;S\left(d_{4}\right)=0.0211$.	$A_{2}>A_{1}>A_{3}>A_{4}$
Our method based on LSF1 (t=3, ρ=1.6)	$S\left(d_{1}\right)=0.0277$$;\;S\left(d_{2}\right)=0.0442$; $S\left(d_{3}\right)=0.0226$$;\;S\left(d_{4}\right)=0.0137$.	$A_{2}>A_{1}>A_{3}>A_{4}$
Our method based on LSF1 (t=3, ε=β=0.5)	$S\left(d_{1}\right)=0.0670$$;\;S\left(d_{2}\right)=0.1262$; $S\left(d_{3}\right)=0.0553$$;\;S\left(d_{4}\right)=0.0339$.	$A_{2}>A_{1}>A_{3}>A_{4}$

**Table 6:** Score functions and ranking orders by different methods.

Methods	$\mathrm{Score}\;\mathrm{Values}\;S\left(d_{i}\right)\left(i=1,2,3,4\right)$	Ranking Orders
Feng et al.’s [35] method based on IVq-RDHLWMSM operator (*k* = 1)	$S\left(d_{1}\right)=1.1187$$;\;S\left(d_{2}\right)=1.2410$; $S\left(d_{3}\right)=1.0981$$;\;S\left(d_{4}\right)=1.0135$	$A_{2}>A_{1}>A_{3}>A_{4}$
Feng et al.’s [35] method based on IVq-RDHLWMSM operator (*k* = 2)	$S\left(d_{1}\right)=0.8789$$;\;S\left(d_{2}\right)=1.1015$; $S\left(d_{3}\right)=0.8146$$;\;S\left(d_{4}\right)=0.6472$	$A_{2}>A_{1}>A_{3}>A_{4}$
Feng et al.’s [35] method based on IVq-RDHLWMSM operator (*k* = 3)	$S\left(d_{1}\right)=0.4962$$;\;S\left(d_{2}\right)=0.6929$; $S\left(d_{3}\right)=0.4163$$;\;S\left(d_{4}\right)=0.2843$	$A_{2}>A_{1}>A_{3}>A_{4}$
Feng et al.’s [35] method based on IVq-RDHLWMSM operator (*k* = 4)	$S\left(d_{1}\right)=0.1901$$;\;S\left(d_{2}\right)=0.3002$; $S\left(d_{3}\right)=0.1426$$;\;S\left(d_{4}\right)=0.0863$	$A_{2}>A_{1}>A_{3}>A_{4}$
Our method based on LSF1(*t* = 3, *k* = 2, *q* = 4)	$S\left(d_{1}\right)=0.0424$$;\;S\left(d_{2}\right)=0.0742$; $S\left(d_{3}\right)=0.0346$$;\;S\left(d_{4}\right)=0.0212$.	$A_{2}>A_{1}>A_{3}>A_{4}$
Our method based on LSF2(t=3, ρ=1.2)	$S\left(d_{1}\right)=0.0358$$;\;S\left(d_{2}\right)=0.0611$; $S\left(d_{3}\right)=0.0297$$;\;S\left(d_{4}\right)=0.0178$.	$A_{2}>A_{1}>A_{3}>A_{4}$
Our method based on LSF3(t=3, ε=0.3,β=0.6)	$S\left(d_{1}\right)=0.0840$$;\;S\left(d_{2}\right)=0.1518$; $S\left(d_{3}\right)=0.0695$$;\;S\left(d_{4}\right)=0.0427$.	$A_{2}>A_{1}>A_{3}>A_{4}$

**Table 7:** The results of Example 4 calculated by different methods.

Methods	$\mathrm{Score}\;\mathrm{Values}\;S\left(d_{i}\right)\left(i=1,2,3,4\right)$	Ranking Orders
Du et al.’s [48] method based on IVPFLWA operator	$S\left(d_{1}\right)=1.5450$$;\;S\left(d_{2}\right)=2.0000$; $S\left(d_{3}\right)=2.2425$$;\;S\left(d_{4}\right)=1.9920$	$A_{3}>A_{4}>A_{2}>A_{1}$
Our method based on LSF1 (*t* = 3, *k* = 2, *q* = 4)	$S\left(d_{1}\right)=0.0442$$;\;S\left(d_{2}\right)=0.0627$; $S\left(d_{3}\right)=0.0822$$;\;S\left(d_{4}\right)=0.0766$.	$A_{3}>A_{4}>A_{2}>A_{1}$

**Table 8:** The final results of Example 4 by the proposed methods (*k* = 2, *q* = 4).

Parameters	$\mathrm{Score}\;\mathrm{Values}\;S\left(d_{i}\right)\left(i=1,2,3,4\right)$	Ranking Orders
Our methos based on LSF1 (*t* = 3)	$S\left(d_{1}\right)=0.0442$$;\;S\left(d_{2}\right)=0.0627$; $S\left(d_{3}\right)=0.0908$$;\;S\left(d_{4}\right)=0.0721$.	$A_{3}>A_{4}>A_{2}>A_{1}$
Our methos based on LSF2 (t=3, ρ=1.6)	$S\left(d_{1}\right)=0.0265$$;\;S\left(d_{2}\right)=0.0339$; $S\left(d_{3}\right)=0.0506$$;\;S\left(d_{4}\right)=0.0408$.	$A_{3}>A_{4}>A_{2}>A_{1}$
Our methos based on LSF3 (t=3, ε=0.6;β=0.4)	$S\left(d_{1}\right)=0.0711$$;\;S\left(d_{2}\right)=0.1136$; $S\left(d_{3}\right)=0.1443$$;\;S\left(d_{4}\right)=0.1191$.	$A_{3}>A_{4}>A_{2}>A_{1}$

**Table 9:** Score values and ranking results of Example 4 with different values of *k* in the IVq-RDHLPWHM operator (*q* = 4).

*k*	$\mathrm{Score}\;\mathrm{Values}\;S\left(d_{i}\right)\left(i=1,2,3,4\right)$	Ranking Orders
*k* = 1	$S\left(d_{1}\right)=0.0462$ $;\;S\left(d_{2}\right)=0.0658$ $;\;S\left(d_{3}\right)=0.0956$ $;\;S\left(d_{4}\right)=0.0791$	$A_{3}>A_{4}>A_{2}>A_{1}$
*k* = 2	$S\left(d_{1}\right)=0.0442$ $;\;S\left(d_{2}\right)=0.0627$ $;\;S\left(d_{3}\right)=0.0908$ $;\;S\left(d_{4}\right)=0.0721$	$A_{3}>A_{4}>A_{2}>A_{1}$
*k* = 3	$S\left(d_{1}\right)=0.0435$ $;\;S\left(d_{2}\right)=0.0617$ $;\;S\left(d_{3}\right)=0.0892$ $;\;S\left(d_{4}\right)=0.0698$	$A_{3}>A_{4}>A_{2}>A_{1}$
*k* = 4	$S\left(d_{1}\right)=0.2093$ $;\;S\left(d_{2}\right)=0.2439$ $;\;S\left(d_{3}\right)=0.2712$ $;\;S\left(d_{4}\right)=0.2278$	$A_{3}>A_{4}>A_{2}>A_{1}$

**Table 10:** The final results of Example 5 by different methods.

Methods	$\mathrm{Score}\;\mathrm{Values}\;S\left(d_{i}\right)\left(i=1,2,3,4\right)$	Ranking Orders
Du et al.’s [48] method based on IVPFLWA operator	Cannot be calculated	-
Our method based on LSF1 (*t* = 3, *k* = 2, *q* = 4)	$S\left(d_{1}\right)=0.0442$$;\;S\left(d_{2}\right)=0.0627$; $S\left(d_{3}\right)=0.0934$$;\;S\left(d_{4}\right)=0.0721$.	$A_{3}>A_{4}>A_{2}>A_{1}$

**Table 11:** Characteristics of different MADM methods.

	Feng et al.’s [35] Method Based on IVq-RDHLWMSM Operator	Du et al.’s [48] Method Based on the IVPFLWA Operator	Our Method Based on the IVq-RDHLPWHM Operator
Allow the sum of MG and NMG to be greater than one	Yes	Yes	Yes
Allow the different semantic gap between adjacent LTs	No	No	Yes
Consider the relationship among multiple attributes	Yes	No	Yes
Reduce the adverse influence of unreasonable evaluation values	No	No	Yes
The degree of flexibility and robustness of the operational rules	Low	Low	High

## Data Availability

All data are included in the manuscript and fully available without restriction.
